# Bacterial Associates of a Gregarious Riparian Beetle With Explosive Defensive Chemistry

**DOI:** 10.3389/fmicb.2018.02361

**Published:** 2018-10-05

**Authors:** Reilly McManus, Alison Ravenscraft, Wendy Moore

**Affiliations:** ^1^Graduate Interdisciplinary Program in Entomology and Insect Science, Tucson, AZ, United States; ^2^Department of Entomology, University of Arizona, Tucson, AZ, United States; ^3^Center for Insect Science, Department of Entomology, University of Arizona, Tucson, AZ, United States

**Keywords:** 16s rDNA, *Spiroplasma*, bombardier beetle, *Brachinus elongatulus*, microbiome, Nematomorpha

## Abstract

Bombardier beetles (Carabidae: Brachininae) are well known for their unique explosive defensive chemistry. These beetles are found in riparian corridors throughout the American Southwest, where they commonly form large diurnal multispecies aggregations in moist areas under rocks, in crevices, and in leaf litter. Using high throughput 16S amplicon sequencing, we provide the first microbiome survey of a bombardier beetle, *Brachinus elongatulus*, collected from two sites in Arizona. Two bacterial taxa were present in all individuals sampled: *Enterococcus* and *Dysgonomonas*. *Enterococcus* has been implicated in the production of fecal aggregation pheromone components, which have been shown to regulate aggregation in the German cockroach; it is possible that *Enterococcus* plays a similar role in *Brachinus*. *Dysgonomonas* was found in all the secretory cells of the defensive system and gut samples. Additional studies are needed to determine if these microbes play a role in these beetles’ unique chemical defense. Results also show that the majority of *B. elongatulus* individuals collected from both sites were infected with *Spiroplasma*. Many *Spiroplasma* are intracellular, vertically transmitted insect symbionts that may manipulate host reproduction (e.g., cause male-killing) or provide resistance to nematodes and/or parasitoid wasps. Defensive protection could be especially beneficial to *B. elongatulus*, which are frequently parasitized by horsehair worms (Nematomorpha). In sum, findings suggest several testable hypotheses on the effects bacteria may have on bombardier beetle behavior and physiology.

## Introduction

**FIGURE 1:**
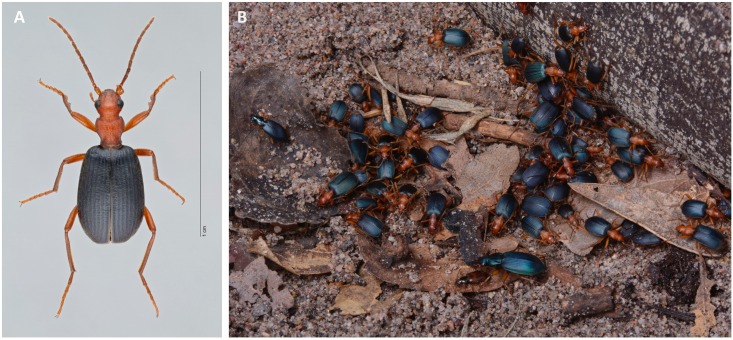
**(A)** Habitus of *Brachinus elongatulus* (UAIC1004027), United States: Arizona, Madera Canyon. **(B)** Multispecies aggregation of carabid beetles photographed in the laboratory after lifting a piece of wood under which the beetles had settled. Individuals were collected along the San Pedro River and are members of the following species: *Brachinus favicollis, B. elongatulus, B. phaeocerus, B. hirsutus, B. lateralis, B. mexicanus, Chlaenius ruficauda, C. cumatilis*, and *Agonum* sp.

**FIGURE 2:**
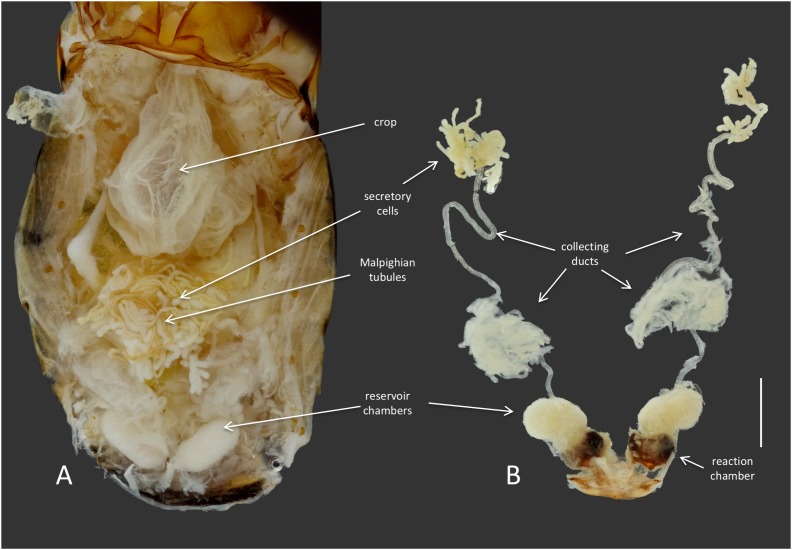
Anatomy of the defensive system of *B. elongatulus* and its orientation inside of the abdomen. **(A)** Dorsal view of the abdomen of *B. elongatulus*, tergites removed. **(B)** Paired defensive gland system dissected from the abdomen. Note that the secretory cells of the defensive system are intertwined with the Malpighian tubules of the digestive system. This bundle lays dorsal to the midgut. Scale bar = 1 mm.

Members of the genus *Brachinus* (Coleoptera: Brachininae) belong to a group of carabids known as bombardier beetles, which produce noxious exothermic defensive sprays to deter their predators (Arndt et al., 2015) (**Figure 1A**). They produce defensive chemicals with a pair of pygidial glands (**Figure 2**). Each gland system consists of secretory cells, a long collecting duct, a reservoir chamber, and a heavily sclerotized reaction chamber. Hydroquinones and hydrogen peroxide are transferred through the collecting ducts and stored in the reservoir chamber. When the beetles are threatened, muscles surrounding the reservoir chamber contract sending these chemical precursors through a one-way valve to mix with catalases and peroxidases in the reaction chamber creating defensive benzoquinones in an exothermic reaction just before they are expelled from the body in an audible explosion (Schildknecht and Holoubek, 1961; Aneshansley et al., 1983). This intricate mechanism is broadly acknowledged as one of the most complex and sophisticated defensive systems known in the animal kingdom (Eisner, 2003).

**FIGURE 3:**
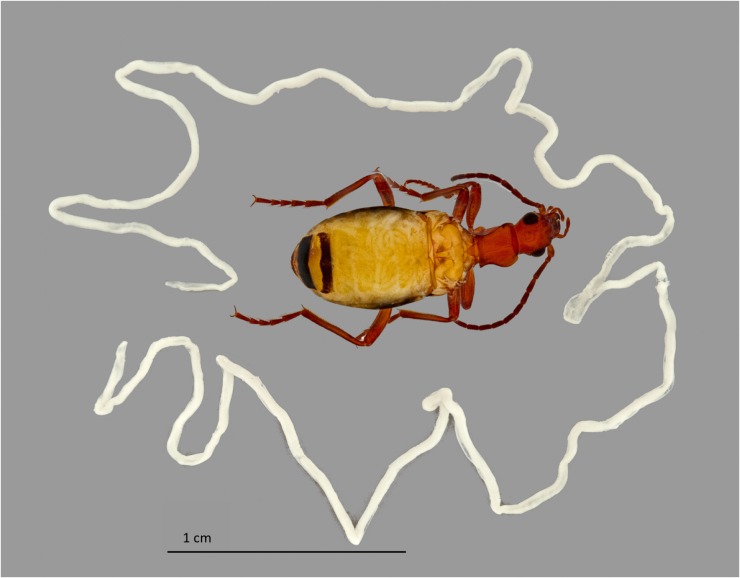
Nematomorphoran infection of *Brachinus elongatulus.* The wings have been removed from the beetle and a horsehair worm can be seen coiled inside the abdomen beneath the abdominal tergites. Another single horsehair worm, removed from another beetle’s abdomen, surrounds the beetle.

Adult *Brachinus elongatulus* are found in moist areas under rocks, in crevices and in leaf litter along riparian corridors in Southeastern Arizona. Females oviposit in the moist mud or gravel along the water’s edge. After hatching, the triungulan larva seeks out a pupa of a water beetle (Hydrophilidae, Dysticidae, Gyrinidae) which it consumes as it undergoes full larval development (Erwin, 1967; Saska and Honek, 2004). Due to living in close proximity to streams, adult *Brachinus* have often been found to be infected with parasitic horsehair worms (Nematomorpha) (McManus and Moore, personal observation) (**Figure 3**).

During the day, adult *Brachinus* are gregarious and commonly form diurnal multispecies aggregations that may contain many species of *Brachinus* as well as members of other carabid genera, such as *Agonum, Chlaenius, Platynus*, and *Galerita* (Schaller et al., in press) (**Figure 1B**). Adults forage at night scavenging on decaying organic matter and acting as opportunistic predators before forming a new aggregation by dawn. Similar to the behavior of some of their gyrinid hosts, *Brachinus* do not return to their aggregation of origin after dispersing at night (Heinrich and Vogt, 1980; Moore, personal observation). Aggregations provide a variety of benefits to the participating individuals, such as providing protection by deterring predators, facilitating mating, and allowing chemically defended organisms to conserve energy (Machado and Vasconcelos, 1998).

Cues involved in the formation of these aggregations are unknown but are thought to be chemical and possibly tactile in nature (Wautier, 1971; Schaller et al., in press). Multispecies aggregations are extremely rare among arthropods with only a few cases being reported in harvestmen (Archnida: Opiliones), net-winged beetles (Coleoptera: Lycidae) and whirligig beetles (Coleoptera: Gyrinidae) (Eisner et al., 1962; Heinrich and Vogt, 1980; Machado and Vasconcelos, 1998).

Other gregarious insects, such as German cockroaches (Ectobiidae), firebrats (Lepismatidae) and desert locusts (Acrididae), have been shown to aggregate in response to volatile fecal pheromones produced by their gut microbiota (Dillion et al., 2000; Woodbury and Gries, 2013; Wada-Katsumata et al., 2015). Digestive microbes in termites have also been implicated in providing cues for nest mate recognition (Minkley et al., 2006). To date the role of microbiota in influencing *Brachinus* aggregation behavior is unknown.

The present study is the first exploration into the bacterial associates of a bombardier beetle and the first survey of a carabid beetle microbiome using high throughput sequencing methods. Previous studies have examined the bacterial communities present in the digestive tracts of three species of carabid beetles—*Harpalus pensylvanicus*, *Anisodactylus sanctaecrucis* (both of which specialize on eating seeds) and *Poecilus chalcites*, a predator of living arthropods. Both of these studies used terminal restriction fragment length polymorphism (tRFLP) in tandem with 16S rRNA gene cloning (Lundgren et al., 2007; Lehman et al., 2009). The objectives of our study were to (1) characterize the microbial communities of *B. elongatulus* using high-throughput 16S amplicon sequencing, (2) determine if there is a core set of sequence variants common across individuals from two localities, (3) determine if *B. elongatulus* hosts any potential obligate or facultative endosymbionts and (4) lay the groundwork for future investigations into how the microbiome of *B. elongatulus* might shape their behavior and/or physiology.

## Materials and Methods

### Insect Collection and Dissections

*Brachinus elongatulus* were collected from two sites in Southeastern Arizona. Thirty-two specimens were sampled from Madera Canyon (Santa Rita Mountains, Santa Cruz Co.) (Site 1) and 5 specimens were sampled from Happy Valley (Rincon Mountains, Pima Co.) (Site 2) (**Table 1**). All specimens were kept alive and transferred back to the lab for dissections. All dissections were performed within 24 h of collection. Specimens were briefly preserved in 100% ethanol and surfaced sterilized using an 8% bleach solution followed by a 1-min wash in phosphate-buffered saline (PBS) and a 1-min wash in 100% ethanol just prior to dissection. Each dissection was made using flame-sterilized forceps and flame-sterilized glass petri dishes. Details on the individuals and tissues sampled are presented in **Table 1**. For 26 specimens from Site 1, the whole bodies, minus the hindwings and elytra, were processed. For an additional six specimens from Site 1, the mid-gut and Malpighian tubules (MGMT), ileum, and the secretory cells of the defensive system were dissected out of the abdomen and processed separately. For the five specimens from Site 2, the MGMT and ileum were dissected out of the abdomen and processed separately.

**Table 1:** 16S Amplicon MiSeq Samples (*Brachinus elongatulus).*

Taxon ID	Unique ID	Body part	Sex	Site-year
Individual_1	DNA4241	Whole body	Female	1-2016
Individual_2	DNA4289	Whole body	Female	1-2016
Individual_3	DNA4500	Whole body	Female	1-2017
Individual_4	DNA4502	Whole body	Female	1-2017
Individual_5	DNA4505	Whole body	Female	1-2017
Individual_6	DNA4507	Whole body	Female	1-2017
Individual_7	DNA4508	Whole body	Female	1-2017
Individual_8	DNA4509	Whole body	Female	1-2017
Individual_9	DNA4520	Whole body	Female	1-2017
Individual_10	DNA4523	Whole body	Female	1-2017
Individual_11	DNA4241	Whole body	Female	1-2017
Individual_12	DNA4241	Whole body	Female	1-2017
Individual_13	DNA4510	Whole body	Male	1-2017
Individual_14	DNA4511	Whole body	Male	1-2017
Individual_15	DNA4512	Whole body	Male	1-2017
Individual_16	DNA4513	Whole body	Male	1-2017
Individual_17	DNA4514	Whole body	Male	1-2017
Individual_18	DNA4515	Whole body	Male	1-2017
Individual_19	DNA4516	Whole body	Male	1-2017
Individual_20	DNA4517	Whole body	Male	1-2017
Individual_21	DNA4518	Whole body	Male	1-2017
Individual_22	DNA4521	Whole body	Male	1-2017
Individual_23	DNA4522	Whole body	Male	1-2017
Individual_24	DNA4532	Whole body	Male	1-2017
Individual_25	DNA4526	Whole body	Male	1-2017
Individual_26	DNA4531	Whole body	Male	1-2017
Individual_27	DNA4249	MGMT	Female	1-2016
	DNA4251	Secretory cells		
	DNA4250	Ileum		
Individual_28	DNA4255	MGMT	Female	1-2016
	DNA4256	Secretory cells		
	DNA4254	Ileum		
Individual_29	DNA4260	MGMT	Female	1-2016
	DNA4259	Secretory cells		
	DNA4258	Ileum		
Individual_30	DNA4274	MGMT	Female	1-2016
	DNA4276	Secretory cells		
	DNA4275	Ileum		
Individual_31	DNA4279	MGMT	Female	1-2016
	DNA4281	Secretory cells		
	DNA4280	Ileum		
Individual_32	DNA4284	MGMT	Female	1-2016
	DNA4286	Secretory cells		
	DNA4285	Ileum		
Individual_33	DNA4228	MGMT	Male	2-2016
	DNA4229	Ileum		
Individual_34	DNA4231	MGMT	Male	2-2016
	DNA4232	Ileum		
Individual_35	DNA4234	MGMT	Male	2-2016
	DNA4235	Ileum		
Individual_36	DNA4237	MGMT	Female	2-2016
	DNA4238	Ileum		
Individual_37	DNA4297	MGMT	Female	2-2016
	DNA4298	Ileum		

*Site 1-2016: Specimens collected from Madera Canyon, AZ (31.74038, −110.88776) in May 2016. Site 1-2017: Specimens collected from Madera Canyon, AZ (31.71299, −110.88776) in September 2017. Site 2-2016: Specimens collected from Happy Valley, AZ (32.15607, −110.47414) in July 2016.*

### DNA Extraction, PCR Amplification, and High-Throughput Sequencing

Dissected tissues and whole bodies were placed in individual 1.5 mL Eppendorf tubes containing lysozyme buffer [180 μl for tissues and 360 μl for whole bodies: 20 mM Tris-HCl (pH 8.0), 2mM EDTA, 1.2% Triton X, 10 mg ml^−1^ lysozyme]. Tissues were ground using sterile DNase/RNase free pestles. Samples were then incubated at 37°C for 90 min. DNA was precipitated and purified following the Qiagen Blood and Tissue Kit protocol for gram-positive bacteria. Using a two-step protocol, the bacterial hypervariable V3-V4 domain of 16S rDNA was PCR amplified using the primers 341F and 785R (Klindworth et al., 2013). The thermocycler protocol was denaturation at 94°C for 3 min followed by 35 cycles of denaturation at 94°C for 45 s, annealing at 50°C for 60 s, and extension at 72°C for 90 s, with a final extension of 72°C for 10 min (Gilbert et al., 2014). PCR products were then purified using QIAquick PCR Purification Kit (Qiagen). In a second PCR unique barcodes were ligated to the amplified region of interest (Hamady et al., 2008). The thermocycler protocol was denaturation at 95°C for 3 min followed by 8 cycles of denaturation at 95°C for 30 s, annealing at 50°C for 30 s, and extension at 68°C for 50 s, with a final extension of 68°C for 10 min. After the second PCR, products were cleaned with magnetic beads (Sera-Mag SpeedBeads, GE Healthcare 65152105050250) and the final concentration of each sample was quantified fluorometrically with a 96-well plate reader (Qubit dsDNA HS kit, Thermo Fisher Q32854) (Rohland and Reich, 2012). The bacterial library was sequenced on an Illumina MiSeq platform (Illumina, San Diego, CA, United States) at The University of Arizona Genomics Core (Tucson, AZ, United States) with 300 × 2 chemistry.

### Illumina Sequence Data Processing

After trimming primer sequences, DADA2 was used to identify sequence variants from raw Illumina paired-end reads (Callahan et al., 2016). DADA2 uses an error model that incorporates quantitative abundances to infer the true abundances of every unique bacterial sequence; it is effectively the same as 100% OTUs but with error removed. Forward and reverse reads were quality filtered, de-replicated, merged, and bimeras were removed. The resulting *de novo* amplicon sequence variants (ASVs) were assigned taxonomic classifications using a native implementation of the RDP classifier (Wang et al., 2007) against version 128 of Silva taxonomic training data formatted for DADA2 (Quast et al., 2013). The R package “phyloseq” (McMurdie and Holmes, 2013) was used to organize, visualize and analyze data. Analyses of whole body individuals from Site 1 samples were rarified to 1669 reads per tissue type. For the analysis of differences in microbial community in organs systems within Site 1 reads were rarified to 1772. For analysis of microbial communities in MGMT and ileum samples from Site 2 reads were rarified to 4468. For comparison of MGMT samples between Site 1 and Site 2 reads were rarified to 3714. For comparison of ileum samples between Site 1 and Site 2 reads were rarified to 1796. All rarefaction depths employed in our analyses adequately sampled these microbial communities (**Supplementary Figure S1**).

### Statistical Analysis

Using the R package “vegan” (Oksanen et al., 2018), non-metric multi-dimensional scaling (NMDS) scaling plot analyses of microbial communities was performed using Bray-Curtis dissimilarity. Permutational analysis of variance (PERMANOVA) was run to test whether within-group distances were significantly different from the between-group distances. PERMANOVA testing was run using the Adonis command. FDR-corrected multiple pairwise *t*-tests were ran in the “phyloseq” (McMurdie and Holmes, 2013) R package using the mt() command.

### *Spiroplasma* Survey Using PCR

DNA extractions from various tissue types of thirty-one previously collected *B. elongatulus* specimens (see **Supplementary Table S1**) were screened for *Spiroplasma* using *Spiroplasma*-diagnostic primers 23f (5′-CTCAGGATGAACGCTGGCGGCAT-3′) and TKSSsp (5′-TAGCCGTGGCTTTCTGGTAA-3′) producing a 410 base pair fragment of the V1-V2 region of 16S rDNA. This primer pair was selected for its ability to amplify almost all *Spiroplasma*, including male-killing and non-male killing strains (Watts et al., 2009). However, these primers can amplify other types of bacteria as well (Watts et al., 2009). The thermocycler protocol was denaturation at 94°C for 2 min followed by 35 cycles of denaturation at 94°C for 30 s, annealing at 50°C for 30 s, and extension at 72°C for 90 s, with a final extension of 72°C for 5 min. PCR products were sequenced with an ABI 3730 at The University of Arizona Genomics Core (Tucson, AZ, United States).

### Molecular Phylogenetic Inference of *Spiroplasma*

We used 16S rDNA to reconstruct the phylogeny of *Spiroplasma*. Five sequences from the *Spiroplasma* PCR survey (410 bp fragment of the V1–V2 region), four ASVs identified as *Spiroplasma* from the Illumina survey (427 bp fragment of the V3–V4 region), two sequences from transcriptomes of *Brachinus elongatulus* from Madera Canyon (available in the Moore lab) (692 bp fragment of the V1–V4 region), and 113 full length 16S Tenericutes sequences from NCBI (DiBlasi et al., 2011; Benson et al., 2012) were used to infer the evolutionary relationships of *Spiroplasma* species.

See **Supplementary Table S2** for accession numbers. The sequences were aligned using MAFFT v 7.310 (Katoh and Standley, 2013) with default settings in Mesquite (Maddison and Maddison, 2017). The aligned matrix was trimmed to the V1–V4 region, resulting in an alignment of 719 bp. The best fitting model of nucleotide evolution was selected with PartitionFinder (Lanfear et al., 2012). The maximum likelihood phylogeny was inferred with the GTR + Gamma model of nucleotide evolution using RAxML v8.2.8 (Stamatakis, 2014) on CIPRES Science Gateway portal (Miller et al., 2010). 500 search replicates were conducted to find the maximum likelihood tree. Clade support was conducted using rapid bootstrapping with a subsequent ML search letting RAxML halt bootstrapping automatically (using MRE-based bootstopping criterion).

## Results

### Data Summary of the MiSeq Analysis

A total of 864,299 rDNA V3–V4 reads were obtained from 54 unique samples comprised of either whole-body individuals or a specific tissue dissected from an individual specimen (**Table 1**). After quality control filtering and merging of paired-end reads 424,425 sequences remained with an average read length of 422 bases. The DADA2 algorithm identified 248 unique ASVs with an average of 26 ASVs per sample. The 54 libraries ranged in size from 1,669 to 54,077 sequences.

### Bacterial Taxa Associated With *Brachinus elongatulus*

#### Site 1: Whole-Body Samples

**FIGURE 4:**
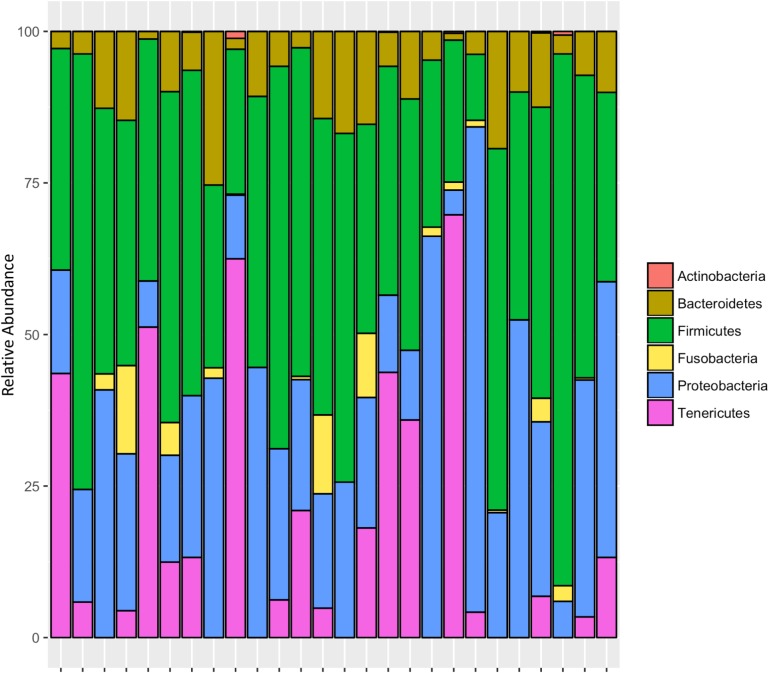
Relative abundance of bacterial phyla in 26 individuals from Site 1 (Madera Canyon, AZ).

After rarifying to 1669 reads per sample, a total of 165 bacterial ASVs across six bacterial phyla were detected in the whole-body samples from Site 1 (Madera Canyon). The bacterial phyla with greater than one percent of total reads across all *B. elongatulus* whole-body individuals were Firmicutes (44%), Proteobacteria (28%), Tenericutes (16%), Bacteroidetes (9%) and Fusobacteria (2%) (**Figure 4**). The top 20 most abundant ASVs are shown in **Table 2**. See **Supplementary Table S3** for a full list of ASVs. There were no significant differences in bacterial communities based on sex (PERMANOVA, *R*^2^ = 0.034, *p* = 0.58) (**Supplementary Figure S2**).

**Table 2:** Taxonomic identities of the 20 most abundant amplicon sequence variants.

ASVid	Avg. (STD)	Phylum	Class	Order	Family	Genus	Accession #
ASV3	16.0 (9.7)	Firmicutes	Bacilli	Lactobacillales	Enterococcaceae	Enterococcus	MH879872
ASV2	12.5 (18.2)	Tenericutes	Mollicutes	Entomoplasmatales	Spiroplasmataceae	Spiroplasma	MH879871
ASV4	10.3 (17.2)	Proteobacteria	γ-proteobacteria	Pseudomonadales	Pseudomonadaceae		MH879873
ASV10	3.8 (3.8)	Firmicutes	Erysipelotrichia	Erysipelotrichales	Erysipelotrichaceae		MH879879
ASV7	3.6 (4.6)	Proteobacteria	γ-proteobacteria	Orbales	Orbaceae		MH879876
ASV11	2.7 (3.9)	Proteobacteria	γ-proteobacteria	Orbales	Orbaceae		MH879880
ASV13	2.5 (5.5)	Firmicutes	Bacilli	Lactobacillales	Lactobacillaceae	Lactobacillus	MH879882
ASV1	2.4 (6.4)	Tenericutes	Mollicutes	Entomoplasmatales	Spiroplasmataceae	Spiroplasma	MH879870
ASV24	2.4 (8.0)	Firmicutes	Bacilli	Lactobacillales	Carnobacteriaceae		MH879893
ASV16	2.2 (4.1)	Fusobacteria	Fusobacteriia	Fusobacteriales	Leptotrichiaceae	Sebaldella	MH879885
ASV15	2.2 (3.9)	Proteobacteria	γ-proteobacteria	Pseudomonadales	Pseudomonadaceae		MH879884
ASV6	2.1 (2.3)	Firmicutes	Bacilli	Lactobacillales			MH879875
ASV28	2.0 (2.3)	Firmicutes	Clostridia	Clostridiales	Ruminococcaceae	Soleaferrea	MH879897
ASV50	1.9 (6.3)	Firmicutes	Bacilli	Lactobacillales	Enterococcaceae	Enterococcus	MH879917
ASV17	1.8 (2.3)	Bacteroidetes	Bacteroidia	Bacteroidales	Porphyromonadaceae	Dysgonomonas	MH879886
ASV9	1.7 (2.1)	Bacteroidetes	Bacteroidia	Bacteroidales	Porphyromonadaceae	Dysgonomonas	MH879878
ASV23	1.7 (3.0)	Bacteroidetes	Bacteroidia	Bacteroidales	Porphyromonadaceae	Dysgonomonas	MH879892
ASV20	1.6 (2.7)	Firmicutes	Bacilli	Lactobacillales	Enterococcaceae		MH879889
ASV14	1.5 (1.5)	Firmicutes	Clostridia	Clostridiales			MH879883
ASV40	1.4 (7.1)	Firmicutes	Bacilli	Lactobacillales	Leuconostocaceae	Weissella	MH879909

*“Avg. (STD)” is the average relative abundance per individual (expressed as a percentage) with the standard deviation in parentheses. Taxonomy was assigned using RDP classifier against the Silva taxonomic training set.*

Six genera were present in at least 50% of the samples from Madera Canyon (**Table 3**). *Enterococcus* and *Dysgonomonas* were present in all 26 whole-body samples. There were no significant differences in *Dysgonomonas* variation between sexes (*t*-test, FDR-corrected *p* = 1.0, **Supplementary Figure S3**). There were also no significant differences in *Enterococcus* variation between sexes (*t*-test, FDR-corrected *p* = 1.0, **Supplementary Figure S3**). *Spiroplasma* was present in 69% of the individuals, *Candidatus* Soleaferrea and *Sulfurospirillum* were present in 65% of the samples, and *Sebaldella* was present in 58% of samples.

**Table 3:** Relative abundance of reads per individual for genera found in at least 50% of the individuals from Site 1 (Madera Canyon, AZ, United States).

	A	B	C	D	E
1	9.2	14.4	51.4		5.9
2	5.6	1.4	10.0		1.9
3	19.2	13.1	34.2	3.6	11.9
4	27.7		40.2	28.8	
5	9.1	8.0	40.5		
6	18.4	8.9	58.6	12.2	
7	9.2	4.5	36.4		1.6
8	54.4	14.7	5.3	3.8	
9	13.1	18.1	52.3	1.3	2.5
10	29.7	1.1	58.6		5.5
11	9.4		59.2		3.9
12	7.5	6.9	74.4	1.9	0.8
13	24.5	6.7	28.9	17.0	
14	28.8	10.6	46.3		3.1
15	27.7		54.4	18.0	
16	17.3		63.3		5.2
17	19.2	4.2	51.7		3.4
18	12.0		78.3	3.8	
19	6.6		76.1	8.0	0.9
20	28.3	10.3	36.4	3.4	3.3
21	26.4	2.3	46.9	0.5	1.7
22	14.8		45.5		
23	22.3	4.1	60.8	6.9	2.0
24	9.5		58.4	8.1	
25	16.4	5.6	75.0	0.3	1.9
26	26.1	5.5	17.0		0.5

*Values range from absent (white), >0 to less than 5% (light blue), >5% to less than 10% (purple), >10% to less than 25% (pink), to >25% (red). A, Dysgonomonas (Bacteroidetes), 29 sequence variants; B, Candidatus Soleaferrea (Firmicutes), 4 sequence variants; C, Enterococcus (Firmicutes), 14 sequence variants; D, Sebaldella (Fusobacteria), 2 sequence variants; E, Sulfurospirillum (Proteobacteria), 1 sequence variant. > 0-5% >5% to less than 10% > 10% to less than 25% Greater than 25%.*

#### Site 1: Gut Tissues and Secretory Cells of the Defensive System

We asked whether the bacterial community differed among organ systems sampled from Site 1. Samples were rarified to 1772 reads per tissue type. PERMANOVA testing resulted in no significant difference in overall bacterial communities between the Malpighian tubules and midgut (MGMT), ileum, and secretory cells of the defensive system (PERMANOVA, *R*^2^ = 0.13, *P* = 0.29; **Supplementary Figure S4**). FDR-corrected pairwise *t*-tests also revealed no significant differences in ASV abundance between tissue types. While it is possible that bacterial communities do not vary between these organ systems, it is also possible that the small sample size did not allow us to detect differences in community composition.

There was a significant difference in overall bacterial communities between MGMT and ileum (PERMANOVA, *R*^2^ = 0.15, *P* = 0.02, **Supplementary Figure S5A**). FDR-corrected multiple pairwise *t*-tests revealed no genera that significantly varied between the tissues. One MGMT sample (DNA4284) was comprised of 100% *Spiroplasma* reads; after removing this sample, there was no longer a significant difference between the two tissue types (PERMANOVA, *R*^2^ = 0.14, *P* = 0.06, **Supplementary Figure S5B**).

**FIGURE 5:**
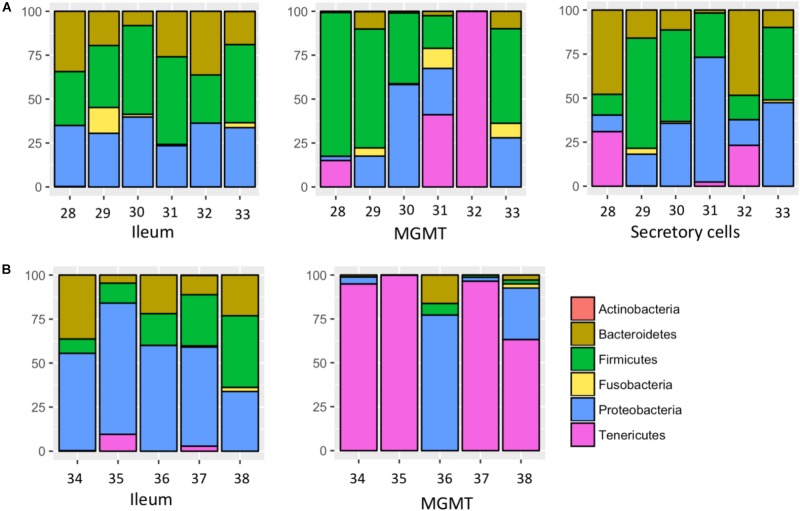
Relative abundance of bacterial phyla in individual tissues from Site 1 **(A)** Madera Canyon, AZ and Site 2 **(B)** Happy Valley, AZ. The Taxon ID number is on the *x*-axis.

MGMT samples had an average of 20 ASVs per tissue type. The bacterial phyla with greater than one percent of total reads across MGMT samples at Site 1 were Firmicutes (44%), Tenericutes (26%), Proteobacteria (22%), Bacteroidetes (4%), and Fusobacteria (4%) (**Figure 5A**). See **Supplementary Table S4** for the top 10 ASVs. The genera *Enterococcus*, *Dysgonomonas*, and *Weisella* were present in four of the five MGMT samples (the fifth sample was composed of 100% S*piroplasma* reads).

The ileum samples had an average of 37 ASVs per tissue type. The bacterial phyla with greater than one percent of total reads across ileum samples Site 1 were Firmicutes (40%), Proteobacteria (33%), Bacteroidetes (24%) and Fusobacteria (3%) (**Figure 5A**). See **Supplementary Table S5** for the top 10 ASVs. For the ileum samples, the genera Candidatus_*Soleaferrea*, *Dysgonomonas*, *Enterococcus*, including ASV3, and *Qingshengfania* were present in all six samples. For the secretory cell samples, the genera *Dysgonomonas*, including ASV9, and *Enterococcus* were present in all samples.

The secretory cell samples had an average of 32 ASVs per tissue type. The bacterial phyla with greater than one percent of total reads across secretory cell samples Site 1 were Firmicutes (34%), Proteobacteria (33%), Bacteroidetes (22%), Tenericutes (9%) and Fusobacteria (1%) (**Figure 5A**). See **Supplementary Table S6** for the top 10 ASVs.

Of note, the genus *Dysgonomonas* was the only genus present in all 6 samples of the secretory cells of the defensive system and in all gut samples.

#### Site 2: Gut Tissues

We asked whether the bacterial community differed among the tissue types sampled from Site 2 (Happy Valley). Samples were rarified to 4468 reads per tissue type. PERMANOVA testing resulted in no significant difference in overall bacterial communities between the MGMT and ileum (PERMANOVA, *R*^2^ = 0.19, *P* = 1.09).

The five MGMT samples had an average of 10 ASVs per sample. The bacterial phyla with greater than one percent of total reads across MGMT samples Site 2 were Tenericutes (71%), Proteobacteria (23%), Bacteroidetes (4%), and Firmicutes (2%) (**Figure 5B**). See **Supplementary Table S7** for the top 10 ASVs for each tissue type No genera were present in every sample.

The five ileum samples had an average of 25 ASVs per sample. The bacterial phyla with greater than one percent of total reads across ileum samples Site 2 were Proteobacteria (56%), Firmicutes (21%), Bacteroidetes (19%), and Tenericutes (3%), (**Figure 5B**). See **Supplementary Table S8** for the top 10 sequence variants for each tissue type. *Dysgonomonas*, *Enterococcus*, including ASV3, and *Pseudomonadaceae* (ASV4) were present in all ileum samples.

### Comparison of Gut Tissues Between Site 1 and Site 2

There were no significant differences in overall bacterial communities in MGMT samples between Site 1 and Site 2 (PERMANOVA, *R*^2^ = 0.13, *P* = 0.13; **Supplementary Figure S6**). There were also no significant differences in overall bacterial communities ileum samples between Site 1 and Site 2 (PERMANOVA, *R*^2^ = 0.14, *P* = 0.06; **Supplementary Figure S6**).

The genera *Dysgonomonas* and *Enterococcus* were present in all ileum samples from both locations. *Spiroplasma* infections were present in individuals from both sites.

### Differences Between Tissue Types Are Consistent With or Without *Spiroplasma*

Since our data suggest that *Spiroplasma* may be symbionts or pathogens of *Brachinus* (see Discussion), we ran all analyses comparing bacterial communities between tissue types twice: once including all *Spiroplasma* reads (the tests reported above), and again omitting all *Spiroplasma* reads to see if the infection was obscuring any signal from the rest of the microbial community. All test results were qualitatively identical, with one small exception: at Site 1, MGMT and ileum differed in community composition when *Spiroplasma* was removed and all samples were included in the test. This difference was driven by the same outlier sample that previously harbored 100% *Spiroplasma* reads.

### Prevalence of *Spiroplasma* in *Brachinus elongatulus*

**FIGURE 6:**
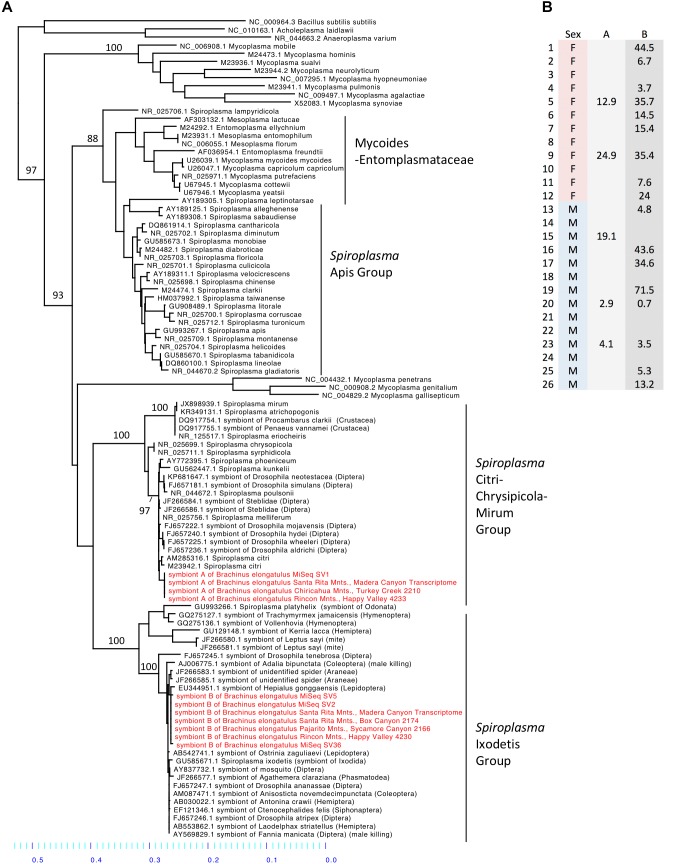
**(A)** Molecular phylogeny of *Spiroplasma.* Sequences from *Brachinus* (MiSeq, PCR survey and transcriptome survey) fall out in two clades (Ixodetis and CCM). **(B)** Relative abundance of *Spiroplasma* in individuals from Site 1. Column A is the relative abundance of *Spiroplasma* symbiont A (svl) belonging to the CCM. Column B is the relative abundance of *Spiroplasma* symbiont B (sv2, sv5, sv36) belonging to the Ixodetis clade.

*Spiroplasma* (phylum Tenericutes) made up 16.2% of the total reads across 26 whole-body individuals. Four ASVs were identified as *Spiroplasma*. All four ASVs were found in individuals from Madera Canyon (ASV1, ASV2, ASV5, ASV36) and three ASVs were found in gut tissues from Happy Valley (ASV1, ASV2, ASV5). In both locations *Spiroplasma* was found in both males and females (**Figure 6B**). There were no significant differences in *Spiroplasma* between sexes (**Supplementary Figure S7**). Some individuals were infected with one sequence variant, while other individuals were infected with two or three ASVs. None of the individuals were infected with all four.

Of the 31 *B. elongatulus* surveyed with *Spiroplasma* specific primers, 29 samples yielded bands and were sent for Sanger sequencing. Twelve samples produced clean sequences. Of those, nine matched *Spiroplasma* and three matched *Streptococcus* or *Pseudomonas* in BLAST searches. Sequences from 17 samples were too noisy to identify, which could suggest that more than one type of bacteria was present (*Spiroplasma* or otherwise). The PCR survey detected the presence of *Spiroplasma* in *B. elongatulus* at an additional three localities (Chiricahua Mountains, Turkey Creek, AZ, Santa Rita Mountains, Box Canyon, AZ, and Parajito Mountains, Sycamore Canyon, AZ) (PCR Table)

### Molecular Phylogenetic Inference of *Spiroplasma*

Sequences from all of our surveys of *Brachinus* fell out into two *Spiroplasma* clades: the Ixodetis clade, and the Citri-Chrysipicola-Mirum (CCM) clade (**Figure 6A** and **Supplementary Figure S8**). The phylogeny indicates there are at least two species of *Spiroplasma* in the *Brachinus elongatulus* populations in Southern Arizona.

## Discussion

Our research on the microbiome of *B. elongatulus* reveals a bacterial community similar to other insects’ microbiota in phylum composition and diversity. In particular, the microbiota of *B. elongatulus* is broadly similar to the microbiota of previously studied carabid beetles. However, our data also show that (1) *Enterococcus* is present in all individuals from two locations, (2) the acid producing *Dysgonomonas* genus is present in all individuals from both locations, and (3) *Spiroplasma*, a common facultative endosymbiont, is present in the majority of individuals from both locations.

### Comparison to Other Insects’ Microbiomes

Among the most abundant phyla present in *B. elongatulus* the Proteobacteria, Firmicutes and Bacteroidetes are also the predominant bacterial phyla detected in gut samples and whole-body samples across several families of Coleoptera (Jones et al., 2013; Yun et al., 2014). In addition, Fusobacteria have been reported in the digestive tract of the predaceous carabid, *P. chalcites* (Lehman et al., 2009). This phylum of bacteria has also been found to be a common gut symbiont of carnivorous birds (Roggenbuck et al., 2014) and is a common hindgut symbiont of cockroaches (Schauer et al., 2012). Fusobacteria have also been found in low abundance in the midgut of several species of mosquitos (Muturi et al., 2017) and in the guts of the dipteran *Bactrocera minax* (Wang et al., 2014), although the functions of these gut bacteria are not known.

Of the six genera present in 50% or more of *B. elongatulus* surveyed, five genera have known associations with insects and, in particular, four of the five genera have associations with fungus-growing termites. A species of *Sebaldella, S. termitidis*, has been isolated and characterized from the gut of a termite (Harmon-Smith et al., 2010). The closest BLAST hit for the *Sulfurospirillum* sequence variant is to an uncultured epsilon-proteobacterium isolated from a gut homogenate of fungus-growing termite, *Macrotermes gilvus.* The closest BLAST hit for all four *Candidatus* Soleaferrea sequence variants is to an uncultured bacterium isolated from the hindgut of cranefly larvae (*Tipula abdominalis*). Only a few species of insects, none of which are carabids, have well characterized microbiomes, therefore, these genera may be more common than we know and/or be more closely related to symbionts of beetles or other non-termite insects that have yet to be surveyed.

Some insects host only a few bacterial species in their guts; for example, fewer than 10 species comprise the core microbiomes of honeybees, reed beetles and fruit flies (Engel and Moran, 2013). Other insects, such as the cockroach and termite, regularly host over 1000 OTUs (operational taxonomic units; roughly equivalent to species) in their gut (Tinker and Ottesen, 2016). *B. elongatulus* had an overall average of 15 ASVs in MGMT samples and an overall average of 31 ASVs in ileum samples. *B. elongatulus* hosts a low bacterial species diversity roughly comparable to that of honeybees and fruit flies. The microbial gut associates of *Brachinus* follow the trend of other insects in that the hindgut hosts more diversity than the midgut. The midgut is often a more hostile environment for microbes to reside due to the suite of digestive and immunological enzymes produced by the midgut epithelium (Douglas, 2015). The ileum environment is often more suitable for microbes, which may benefit from the ion and metabolite rich filtrate delivered into the hindgut by the Malpighian tubules.

Previous studies (Lundgren et al., 2007; Lehman et al., 2009) have examined the microbial communities present in digestive tracts of three species of carabid beetles with different diets: two granivores- *Harpalus pensylvanucus* and *Ansiodactylus sanctaecrucis* and one predator, *Poecilus chalcites.* All three species, like *B. elongatulus*, had an abundance of Proteobacteria. The community of *B. elongatulus* described here and the other predator, *P. chalcites* had the most similar bacterial community compositions with the most abundant OTUs being represented by Firmicutes, Fusobacteria, Proteobacteria and Bacteroidetes. The genera *Enterococcus* and *Weissella* were also identified in *P. chalcites.* It is impossible to more comprehensively compare the microbiomes of these other carabids to that of *B. elongatulus* due to the differences in experimental approach.

### The Genus *Enterococcus* Was Present in All Individuals

*Enterococcus* and six other genera of the lactic-acid bacterial order Lactobacillales were present in *B. elongatulus*. Interestingly, a single ASV of *Enterococcu*s (ASV3) was present in every ileum sample from both sites and in 88% of whole body samples from Site 1. *Enterococcus* is a common inhabitant of the digestive tract of mammals, birds, reptiles, and insects. It has been found across a broad range of insect orders with the most prevalent species studied being *E. faecalis*, *E. faecium*, *E. casseliflavus* (Martin and Mundt, 1972) and most recently *E. termitis*, which was isolated from the gut of a termite (Svec et al., 2006). *E. faecalis* has been shown to support digestion in the carabid, *Harpalus pensylvanicus* (Schmid et al., 2014). *E. casseliflavus* has been implicated in assisting Lepidopteran larvae metabolize toxic compounds ingested from plants, for example by forming a monospecific biofilm in the foregut of *Hyles euphorbiae* (Vilanova et al., 2016).

Presence of a single ASV in most individuals from two localities suggests the potential for a specific symbiotic association between *Brachinus* and this *Enterococcus* species. Our data cannot confirm this, nor can they indicate the potential function of such a relationship. However, some species of these bacteria have been implicated in the production of volatile carboxylic acids (VCAs) that act as fecal aggregation pheromone components, which regulate aggregation in the German cockroach (Wada-Katsumata et al., 2015). Cockroach feces containing isolates of *Enterococcus avium* were found to have a significant effect on German cockroach aggregation compared to axenic cockroach feces. Isolates of *Enterobacter cloacae* were found to cause an aggregation response in the firebrat, *Thermobia domestica* (Woodbury and Gries, 2013). *Brachinus* are notable for their multispecies aggregations and it remains unknown what triggers them. Future research should investigate whether volatiles produced by *Enterococcus* induce aggregation, not only in *B. elongatulus*, but also in other *Brachinus* species.

### The Genus *Dysgonomonas* Was Present in All Individuals

The genus *Dysgonomonas* was present in all individuals surveyed although, unlike *Enterococcus*, there was not a single common ASV. Indeed, *Dysgonomonas* accounted for the most sequence variants assigned to a single genus (28 ASVs). *Dysgonomonas* has been found in the digestive tracts of several insects with varying diets, such as fungus-growing termites (*Macrotermes annadalei*), subterranean termites *Reticulitermes speratus*, and several *Drosophila* populations (Chandler et al., 2011; Yang et al., 2014; Pramono et al., 2015). Within Coleoptera, *Dysgonomonas* has been found to be highly abundant in two species of dung beetle (Scarabaeidae) with different dietary lifestyles- detritivore, *Pachysoma endroedyi*, and dry-dung-feeding, *P. striatum* (Franzini et al., 2016). *Dysgonomonas* has also been found in the digestive tracts of red palm weevil larvae, *Rhynchophorus ferrugineus* (Dryophthoridae) (Muhammad et al., 2017). Two species of *Dysgonomonas* from the guts of the termites have been characterized and both species ferment glucose and xylan as a sole carbon source to produce acetic acid as the major end product (Yang et al., 2014; Pramono et al., 2015). Other species of *Dysgonomonas* have been found to produce lactic acid, propionic acid, succinic acid, and/or acetic acid as the major end product of glucose fermentation (Sakamoto, 2014).

*Dysgonomonas* was found in all of the *Brachinus* digestive tract samples, but more interestingly it was the only genus present in all six defensive secretory cell samples. Additional studies are needed to determine if these microbes play a role in these beetles’ unique chemical defense. However, we noted that *Brachinus* has a unique anatomical association between the defensive and digestive systems, that has apparently not been reported from other carabid beetles. The secretory cells of the defensive system are always intertwined with the Malpighian tubules of the digestive system (**Figure 2**).

It seems that proximity to the digestive and defensive systems is important. In fact, during dissections of individuals with late-stage nematomorphan infections, we have observed the secretory cells adhered to the ileum. In these cases, the Malpighian tubules were no longer present, likely having been consumed by the parasite. The secretory cells of the defensive system, which are thought to produce precursors of quinones used in bombardier beetle defense, have a high density of mitochondria and are very metabolically active (Di Giulio et al., 2015). It could be that the association with the digestive system is important solely for detoxification. It is possible that the association is also important for secretory cells to obtain nutrients and/or building blocks for quinone synthesis. The close association between the defensive system and the Malpighian tubules is most likely due to the need for detoxification. Our data cannot confirm the involvement of microbes in these processes, more studies are needed to determine if microbes play a role.

### *Spiroplasma* Was Present in the Majority of Individuals

*Spiroplasma* accounted for 16.2% of the microbiota of *B. elongatulus*, and the genus was present in 70% of individuals surveyed. In *B. elongatulus*, we hypothesize that at least one species of *Spiroplasma* is an intracellular symbiont and/or present in the hemolymph because the bacterium was successfully amplified from the muscle tissue in an individual leg. *Spiroplasma* are estimated to infect at least 7% of all insects (Duron et al., 2008). *Spiroplasma* have been found to have commensal, mutualistic and/or pathogenic relationships with their plant and arthropod hosts (e.g., crustaceans, arachnids, insects). Insects most commonly infected with *Spiroplasma* include species of Odonata, Hemiptera, Hymenoptera, Diptera, Lepidoptera and Coleoptera (Cisak et al., 2015). In Coleoptera, three species of *Spiroplasma* have been characterized from members of Scarabaeidae, Lampyridae, and Cantharidae (Whitcomb et al., 1993; Hackett et al., 1996). *Spiroplasma* has also been found in Tenebrionidae and Coccinellidae (Tinsley and Majerus, 2006; Wang and Zhang, 2015). Different species of *Spiroplasma* have been shown (1) to be pathogens of honeybees (Schwarz et al., 2014), (2) to cause reproductive manipulation in planthoppers and *Drosophila* (Haselkorn, 2010; Sanada-Morimura et al., 2013), (3) to provide host defense against nematode-induced sterilization in *Drosophila neotestacea* (Jaenike et al., 2010) and parasitoid wasps (Xie et al., 2014), and (4) there are many *Spiroplasma*-host interactions in a wide variety of insects, crustaceans, and arachnids that are not well characterized (Cisak et al., 2015). Future research is needed to determine whether *Spiroplasma* are mutualists, pathogens, or commensals in *B. elongatulus*.

*Spiroplasma* sequences from the MiSeq, PCR and transcriptome surveys fell out in two separate clades (Ixodetis and CCM), therefore it appears at least two species are present. It was not possible to derive the function of these *Spiroplasma* species based on their positions in the phylogeny. The CCM clade contains plant and crustacean pathogens, male-killing species of dipteran hosts, as well as a recently identified species that encodes a ribosome-inactivating protein (RIP) that has defensive effects against nematode infection in *Drosophila neotestacea* (Hamilton et al., 2016). The Ixodetis clade houses male-killing species of coleopteran, dipteran and lepidopteran hosts, defensive species that protect *Drosophila* against parasitoid wasps (Xie et al., 2014) and aphids against pathogenic fungi (Lukasik et al., 2013), and this clade also house a suite of commensal species and species with unknown function (DiBlasi et al., 2011).

We found *Brachinus* that were doubly (*N* = 7) infected with *Spiroplasma* ASVs. Co-infection by two or more species of *Spiroplasma* in arthropods hosts has also been reported in Hymenoptera. Two pathogenic species of *Spiroplasma* belonging to the Apis clade, *S. apis* and *S. melliferum*, have been found to co-infect honeybee colonies in North and South America (Schwarz et al., 2014). Due to the presence of *Spiroplasma* in male and female *B. elongatulus*, we hypothesize our *Spiroplasma* species do not cause male killing (**Figure 6B**), parthenogenesis or feminization. Because of the difficulty in rearing these beetles in captivity and performing controlled matings with newly enclosed adults (the larvae are parasitoids of diving beetles), we are unlikely to be able to rule out cytoplasmic incompatibility (CI), although CI has not yet been associated with *Spiroplasma*. A more likely possibility is a defensive role. *Brachinus elongatulus* live in riparian areas and are commonly found infected with nematomorpha (McManus and Moore, personal observation). Further investigation is needed to determine if one or both of the species of *Spiroplasma* confer defense against helminth parasite infection.

## Conclusion

This study paves the way for further investigations into the roles of microorganisms in the behavior, immune function, and physiology of *B. elongatulus.* In particular, our results suggest that a single sequence variant of *Enterococcus*, the genus *Dysgonomonas*, and the genus *Spiroplasma* may have specialized relationships with *Brachinus* that merit further investigation. To resolve the number of *Spiroplasma* infecting *B. elongatulus* and to derive their function, genome sequencing from both cultivable and uncultivable associates would provide important insight. Fluorescence *in situ* hybridization (FISH) could provide insight into localizing and confirming the presence of *Dysgonomonas.* Microbial cultures of *Brachinus* feces, treatment of beetles with antibiotics and follow-up behavioral studies could also provide insight into the potential roles bacteria may be playing in *Brachinus* aggregation behavior.

## Data Accessibility

Sequences and associated metadata are archived under NCBI BioProject PRJNA489455.

## Author Contributions

RM designed the experiments, performed the fieldwork, processed the samples, analyzed the data, and prepared the manuscript. AR processed the samples, analyzed the data, and prepared the manuscript. WM designed the experiments, processed the samples, analyzed the data, and prepared the manuscript.

## Conflict of Interest Statement

The authors declare that the research was conducted in the absence of any commercial or financial relationships that could be construed as a potential conflict of interest.
