# Supplementary material for: Bacterial Associates of a Gregarious Riparian Beetle With Explosive Defensive Chemistry
Source: Front Microbiol. 2018 Oct 5;9:2361. doi: 10.3389/fmicb.2018.02361 (PMC6182187; doi:10.3389/fmicb.2018.02361)
Supplement: Supplementary file 9 [file Table_1.docx]

**Table S1**: **PCR Survey of *Brachinus elongatulus* with *Spiroplasma* specific primers**

Specimens used in *Spiroplasma* PCR survey that sequenced successfully.

| Unique ID | Body part | Sex | Collection Site | Latitude | Longitude | Year | Accession # |
| --- | --- | --- | --- | --- | --- | --- | --- |
| DNA4230 | Internal organs, excluding ileum, MGMT | Male | Rincon Mnts.,  Happy Valley | 32.15607 | -110.474 | Jul 2016 | MH880086 |
| DNA4233 | Internal organs, excluding ileum, MGMT | Male | Rincon Mnts.,  Happy Valley | 32.15607 | -110.474 | Jul 2016 | MH880089 |
| DNA2210 | Right middle leg | Undet | Chiricahua Mnts, Turkey Creek | 31.85077 | -109.325 | May 2010 | MH880088 |
| DNA4372 | Right middle leg | Male | Santa Rita Mnts., Madera Canyon | 31.74038 | -110.887 | Mar 2017 | MH880090 |
| DNA4283 | Fat body | Female | Santa Rita Mnts., Madera Canyon | 31.712994 | -110.873 | Sept 2016 | MH880091 |
| DNA4375 | Right middle leg | Female | Santa Rita Mnts., Madera Canyon | 31.712994 | -110.873 | Sept 2016 | MH880092 |
| DNA4288 | Fat body | Female | Santa Rita Mnts., Madera Canyon | 31.712994 | -110.873 | Sept 2016 | MH880093 |
| DNA4287 | Ovaries | Female | Santa Rita Mnts., Madera Canyon | 31.712994 | -110.873 | Sept 2016 | MH880094 |
| DNA2174 | Right middle leg | Undet | Santa Rita Mnts.,  Box Canyon | 31.7989 | -110.771 | Jun 2009 | MH880085 |
| DNA2172 | Right middle leg | Undet | Parajito Mnts., Sycamore Canyon | 31.43194 | -111.188 | Mar 2010 | MH880087 |
| DNA2173 | Right middle leg | Undet | Parajito Mnts., Sycamore Canyon | 31.43194 | -111.188 | Mar 2010 | MH880095 |
