# Supplementary material for: Bacterial Associates of a Gregarious Riparian Beetle With Explosive Defensive Chemistry
Source: Front Microbiol. 2018 Oct 5;9:2361. doi: 10.3389/fmicb.2018.02361 (PMC6182187; doi:10.3389/fmicb.2018.02361)
Supplement: Supplementary file 10 [file Table_2.docx]

**Table S2: Accession numbers used to infer molecular phylogeny of *Spiroplasma****.* Accession numbers from NCBI of the 16S rDNA sequences included in tree.

| **Clade** | **Taxon** | **Accession #** |
| --- | --- | --- |
| Outgroup | *Bacillus subtilis* | HQ228563 |
| Mycoides-Entomoplasmataceae (ME) | Mycoplasma neurolyticum | M23944 |
|  | Mycoplasma sualvi | M23936 |
|  | Mycoplasma pulmonis | M23941 |
|  | Mycoplasma hominis | M24473 |
|  | Mycoplasma synoviae | X52083 |
|  | Entomoplasma freundtii | AF036954 |
|  | Mesoplasma lactucae | AF303132 |
|  | Mycoplasma sp. | M24478 |
|  | Mesoplasma entomophilum | M23931 |
|  | Mycoplasma ellychnium | M24292 |
|  | Mycoplasma mycoides | U26039 |
|  | Mycoplasma capricolum | U26047 |
|  | Mycoplasma putrefaciens | NR_025971 |
|  | Mycoplasma cottewii | U67945 |
|  | Mycoplasma yeatsii | U67946 |
| Apis | S. culicicola (Diptera) | NR_025701 |
|  | S. chinense (plants) | NR_025698 |
|  | S. velocicrescens (Hymenoptera) | AY189311 |
|  | S. velocicrescens (Hymenoptera) | NR_025713 |
|  | S. diabroticae (Coleoptera) | GU908490 |
|  | S. monobiae (Hymenoptera) | GU585673 |
|  | S. cantharicola (Coleoptera) | DQ861914 |
|  | Spiroplasma sp. (plants) | EF151267 |
|  | S. tabanidicola (Diptera) | GU585670 |
|  | S. lineolae (Diptera) | DQ860100 |
|  | S. gladiatoris (Diptera) | M24475 |
|  | S. litorale (Diptera) | GU908489 |
|  | S. turonicum (Diptera) | NR_025712 |
|  | S. corruscae (Coleoptera) | NR_025700 |
|  | S. apis (Hymenoptera) | GU993267 |
|  | S. montanense (Diptera) | NR_025709 |
|  | S. taiwanense (Diptera) | HM037992 |
|  | S. leptinotarasae (Coleoptera) | AY189305 |
|  | S. alleghenen (Mecoptera) | AY189125 |
|  | S. sabaudiense (Diptera) | AY189308 |
| Citri-Chrysopicola-Mirum (CCM) | Procambarus clarkia (Decapoda) | DQ917754 |
|  | Panaeus vannamei (Decapoda) | DQ917755 |
|  | S. mirum (Decapoda) | DQ917756 |
|  | Tabanidae sp. (Diptera | EF491664 |
|  | Tababus atratus (Diptera) | AY189314 |
|  | Haematopota sp. (Diptera) | EF491665 |
|  | S. chrysopicola (Diptera) | NR_025699 |
|  | S. syrphidicola (Diptera) | NR_025711 |
|  | S.citri (plants) | M23942 |
|  | Spinturnix sp. (Mesostigmata) | DQ28984 |
|  | S. phoeniceum (plants) | AY772395 |
|  | S. kunkelii (plants) | GU562447 |
|  | Drosophila mojavensis (Diptera) | FJ657217 |
|  | Drosophila mojavensis (Diptera) | FJ657222 |
|  | Drosophila hydei (Diptera) | FJ657240 |
|  | Drosophila wheeleri (Diptera) | FJ657225 |
|  | Drosophila aldrichi (Diptera) | FJ657236 |
|  | Hippoboscoidea, Streblidae (Diptera) | JF266584 |
|  | Hippoboscoidea, Streblidae (Diptera) | JF266586 |
|  | S. melliferum | NR_025756 |
|  | S. insolitum | NR_025705 |
|  | Drosophila willistoni (Diptera) | M24483 |
|  | Drosophila simulans (Diptera) | FJ657181 |
| Ixodetis | Laelapidae mite (Mesostigmata) | JF266582 |
|  | Neriene clathrata (Araneae) | EU727102 |
|  | Tetragnatha montana (Araneae) | EU727105 |
|  | Meta segmentata (Araneae) | EU727104 |
|  | Meta mengei (Araneae) | EU727103 |
|  | Hepialus gonggaensis (Lepidoptera) | EU344951 |
|  | Araneus diadematus (Araneae) | EU727099 |
|  | Chrysolina varians (Coleoptera) | EU727100 |
|  | Adalia bipunctata (Coleoptera) | AJ006775 |
|  | S. ixodetis (Ixodida) | GU585671 |
|  | Laodelphax striatellus (Hemiptera) | AB553862 |
|  | Antonina crawii (Hemiptera) | AB030022 |
|  | Agathemera claraziana (Phasmatodea) (A33 gut) | JF266577 |
|  | Drosophila ananassae (Diptera) | FJ657247 |
|  | Ctenocephalides felis (Siphonaptera) | EF121346 |
|  | Anisosticta novemdecimpunctata (Coleoptera) | AM087471 |
|  | Fannia manicata (Diptera) | AY569829 |
|  | Drosophila atripex (Diptera) | FJ657246 |
|  | Drosophila ananassae (Diptera) | FJ657248 |
|  | Notostira elongata (Hemiptera) | EU727098 |
|  | environmental sample | AY837732 |
|  | Anopheles sp. (Diptera) | AY837733 |
|  | Anopheles sp. (Diptera) | AY837731 |
|  | Ostrinia zaguliaevi (Lepidoptera) | AB542741 |
|  | unidentified spider (Araneae) | JF266583 |
|  | unidentified spider (Araneae) | JF266585 |
|  | Tipula oleracea (Diptera) | EU727101 |
|  | Drosophila tenebrosa (Diptera) | FJ657245 |
|  | S. platyhelix (Odonata) | DQ860101 |
|  | S. platyhelix (Odonata) | GU993266 |
|  | Trachymyrmex jamaicensis (Hymenoptera) | GQ275127 |
|  | Vollenhovia sp. (Hymenoptera) | GQ275136 |
|  | Kerria lacca (Hemiptera) | GU129148 |
|  | Leptus sayi (Trombidiformes) (Host A33) | JF266580 |
|  | Leptus sayi (Trombidiformes) (Host A78) | JF266581 |
