# Supplementary material for: Bacterial Associates of a Gregarious Riparian Beetle With Explosive Defensive Chemistry
Source: Front Microbiol. 2018 Oct 5;9:2361. doi: 10.3389/fmicb.2018.02361 (PMC6182187; doi:10.3389/fmicb.2018.02361)
Supplement: Supplementary file 11 [file Table_3.docx]

**Table S3**: **Taxonomic identities of the 165 amplicon sequence variants from whole bodies from Site 1 (Madera Canyon, AZ).** “Avg. (STD)” is the average relative abundance per individual with the standard deviation in parentheses. Taxonomy was assigned using RDP classifier against the Silva taxonomic training set.

| **ASVid** | **Avg. (STD)** | **Phylum** | **Class** | **Order** | **Family** | **Genus** | **Accession #** |  |  |
| --- | --- | --- | --- | --- | --- | --- | --- | --- | --- |
| ASV3 | 16.0 (9.7) | Firmicutes | Bacilli | Lactobacillales | Enterococcaceae | Enterococcus | MH879872 |  |  |
| ASV2 | 12.5 (18.2) | Tenericutes | Mollicutes | Entomoplasmatales | Spiroplasmataceae | Spiroplasma | MH879871 |  |  |
| ASV4 | 10.3 (17.2) | Proteobacteria | γ-proteobacteria | Pseudomonadales | Pseudomonadaceae |  | MH879873 |  |  |
| ASV10 | 3.8 (3.8) | Firmicutes | Erysipelotrichia | Erysipelotrichales | Erysipelotrichaceae |  | MH879879 |  |  |
| ASV7 | 3.6 (4.6) | Proteobacteria | γ-proteobacteria | Orbales | Orbaceae |  | MH879876 |  |  |
| ASV11 | 2.7 (3.9) | Proteobacteria | γ-proteobacteria | Orbales | Orbaceae |  | MH879880 |  |  |
| ASV13 | 2.5 (5.5) | Firmicutes | Bacilli | Lactobacillales | Lactobacillaceae | Lactobacillus | MH879882 |  |  |
| ASV1 | 2.4 (6.4) | Tenericutes | Mollicutes | Entomoplasmatales | Spiroplasmataceae | Spiroplasma | MH879870 |  |  |
| ASV24 | 2.4 (8.0) | Firmicutes | Bacilli | Lactobacillales | Carnobacteriaceae |  | MH879893 |  |  |
| ASV16 | 2.2 (4.1) | Fusobacteria | Fusobacteriia | Fusobacteriales | Leptotrichiaceae | Sebaldella | MH879885 |  |  |
| ASV15 | 2.2 (3.9) | Proteobacteria | γ-proteobacteria | Pseudomonadales | Pseudomonadaceae |  | MH879884 |  |  |
| ASV6 | 2.1 (2.3) | Firmicutes | Bacilli | Lactobacillales |  |  | MH879875 |  |  |
| ASV28 | 2.0 (2.3) | Firmicutes | Clostridia | Clostridiales | Ruminococcaceae | Soleaferrea | MH879897 |  |  |
| ASV50 | 1.9 (6.3) | Firmicutes | Bacilli | Lactobacillales | Enterococcaceae | Enterococcus | MH879917 |  |  |
| ASV17 | 1.8 (2.3) | Bacteroidetes | Bacteroidia | Bacteroidales | Porphyromonadaceae | Dysgonomonas | MH879886 |  |  |
| ASV9 | 1.7 (2.1) | Bacteroidetes | Bacteroidia | Bacteroidales | Porphyromonadaceae | Dysgonomonas | MH879878 |  |  |
| ASV23 | 1.7 (3.0) | Bacteroidetes | Bacteroidia | Bacteroidales | Porphyromonadaceae | Dysgonomonas | MH879892 |  |  |
| ASV20 | 1.6 (2.7) | Firmicutes | Bacilli | Lactobacillales | Enterococcaceae |  | MH879889 |  |  |
| ASV14 | 1.5 (1.5) | Firmicutes | Clostridia | Clostridiales |  |  | MH879883 |  |  |
| ASV40 | 1.4 (7.1) | Firmicutes | Bacilli | Lactobacillales | Leuconostocaceae | Weissella | MH879909 |  |  |
| ASV5 | 1.2 (4.3) | Tenericutes | Mollicutes | Entomoplasmatales | Spiroplasmataceae | Spiroplasma | MH879874 |  |  |
| ASV29 | 1.0 (5.0) | Proteobacteria | γ-proteobacteria | Pseudomonadales | Pseudomonadaceae | Pseudomonas | MH879898 |  |  |
| ASV35 | 0.9 (1.4) | Proteobacteria | ε-proteobacteria | Campylobacterales | Campylobacteraceae | Sulfurospirillum | MH879904 |  |  |
| ASV26 | 0.8 (1.6) | Firmicutes | Bacilli | Lactobacillales |  |  | MH879895 |  |  |
| ASV46 | 0.8 (1.9) | Firmicutes | Bacilli | Lactobacillales | Enterococcaceae | Enterococcus | MH879919 |  |  |
| ASV52 | 0.8 (1.6) | Firmicutes | Bacilli | Lactobacillales | Enterococcaceae | Enterococcus | MH879919 |  |  |
| ASV56 | 0.8 (2.7) | Proteobacteria | γ-proteobacteria | Pseudomonadales | Pseudomonadaceae |  | MH879923 |  |  |
| ASV30 | 0.7 (1.2) | Firmicutes | Erysipelotrichia | Erysipelotrichales | Erysipelotrichaceae |  | MH879899 |  |  |
| ASV42 | 0.6 (0.7) | Bacteroidetes | Bacteroidia | Bacteroidales | Porphyromonadaceae | Dysgonomonas | MH879911 |  |  |
| ASV72 | 0.5 (1.0) | Firmicutes | Erysipelotrichia | Erysipelotrichales | Erysipelotrichaceae |  | MH879939 |  |  |
| ASV64 | 0.5 (0.7) | Proteobacteria | α-proteobacteria | Rhizobiales | Beijerinckiaceae | Qingshengfania | MH879931 |  |  |
| ASV82 | 0.5 (1.5) | Proteobacteria | γ-proteobacteria | Pseudomonadales | Pseudomonadaceae |  | MH879949 |  |  |
| ASV37 | 0.4 (0.6) | Bacteroidetes | Bacteroidia | Bacteroidales | Porphyromonadaceae | Dysgonomonas | MH879906 |  |  |
| ASV88 | 0.4 (1.5) | Firmicutes | Bacilli | Lactobacillales | Enterococcaceae | Enterococcus | MH879955 |  |  |
| ASV79 | 0.4 (2.0) | Firmicutes | Bacilli | Lactobacillales | Carnobacteriaceae |  | MH879946 |  |  |
| ASV78 | 0.4 (0.8) | Firmicutes | Bacilli | Lactobacillales |  |  | MH879945 |  |  |
| ASV45 | 0.3 (0.5) | Bacteroidetes | Bacteroidia | Bacteroidales | Porphyromonadaceae | Dysgonomonas | MH879914 |  |  |
| ASV96 | 0.3 (1.3) | Proteobacteria | α-proteobacteria | Rhizobiales | Rhizobiaceae | Rhizobium | MH879963 |  |  |
| ASV33 | 0.3 (0.7) | Firmicutes |  |  |  |  | MH879902 |  |  |
| ASV65 | 0.3 (0.9) | Proteobacteria | β-proteobacteria | Neisseriales | Neisseriaceae |  | MH879932 |  |  |
| ASV41 | 0.3 (1.3) | Proteobacteria | γ-proteobacteria | Enterobacteriales | Enterobacteriaceae |  | MH879910 |  |  |
| ASV70 | 0.3 (0.6) | Firmicutes |  |  |  |  | MH879937 |  |  |
| ASV19 | 0.3 (0.6) | Proteobacteria | γ-proteobacteria | Enterobacteriales | Enterobacteriaceae |  | MH879888 |  |  |
| ASV109 | 0.3 (1.5) | Bacteroidetes | Bacteroidia | Bacteroidales | Porphyromonadaceae | Dysgonomonas | MH879975 |  |  |
| ASV47 | 0.3 (0.9) | Proteobacteria | γ-proteobacteria | Orbales | Orbaceae | Gilliamella | MH879915 |  |  |
| ASV87 | 0.3 (0.6) | Proteobacteria | γ-proteobacteria | Xanthomonadales | Xanthomonadaceae | Wohlfahrtiimonas | MH879954 |  |  |
| ASV22 | 0.3 (1.0) | Proteobacteria | γ-proteobacteria | Enterobacteriales | Enterobacteriaceae |  | MH879891 |  |  |
| ASV31 | 0.3 (0.5) | Firmicutes | Clostridia | Clostridiales |  |  | MH879900 |  |  |
| ASV60 | 0.3 (0.9) | Firmicutes | Clostridia | Clostridiales | Lachnospiraceae | Tyzzerella_3 | MH879927 |  |  |
| ASV101 | 0.3 (1.3) | Firmicutes |  |  |  |  | MH879968 |  |  |
| ASV142 | 0.2 (1.3) | Firmicutes | Bacilli | Lactobacillales | Carnobacteriaceae | Carnobacterium | MH880007 |  |  |
| ASV111 | 0.2 (1.2) | Firmicutes | Bacilli | Lactobacillales | Enterococcaceae | Enterococcus | MH879977 |  |  |
| ASV105 | 0.2 (0.6) | Bacteroidetes | Bacteroidia | Bacteroidales | Porphyromonadaceae | Dysgonomonas | MH879971 |  |  |
| ASV75 | 0.2 (0.7) | Proteobacteria | γ-proteobacteria | Orbales | Orbaceae |  | MH879942 |  |  |
| ASV44 | 0.2 (0.6) | Firmicutes | Clostridia | Clostridiales | Ruminococcaceae | Anaerotruncus | MH879913 |  |  |
| ASV12 | 0.2 (0.4) | Proteobacteria | γ-proteobacteria | Pseudomonadales | Pseudomonadaceae |  | MH879881 |  |  |
| ASV49 | 0.2 (0.4) | Proteobacteria | β-proteobacteria | Burkholderiales | Comamonadaceae |  | MH880059 |  |  |
| ASV55 | 0.2 (0.5) | Proteobacteria | γ-proteobacteria | Orbales | Orbaceae | Schmidhempelia | MH879922 |  |  |
| ASV68 | 0.2 (0.5) | Proteobacteria | γ-proteobacteria | Orbales | Orbaceae | Gilliamella | MH879935 |  |  |
| ASV73 | 0.2 (0.7) | Proteobacteria | γ-proteobacteria | Pseudomonadales | Pseudomonadaceae |  | MH879940 |  |  |
| ASV74 | 0.2 (0.4) | Bacteroidetes | Bacteroidia | Bacteroidales | Porphyromonadaceae | Dysgonomonas | MH879941 |  |  |
| ASV81 | 0.2 (0.8) | Bacteroidetes | Bacteroidia | Bacteroidales | Porphyromonadaceae | Dysgonomonas | MH879948 |  |  |
| ASV119 | 0.2 (0.5) | Proteobacteria | γ-proteobacteria | Xanthomonadales | Xanthomonadaceae | Wohlfahrtiimonas | MH879985 |  |  |
| ASV120 | 0.2 (0.9) | Proteobacteria | γ-proteobacteria | Xanthomonadales | Xanthomonadaceae | Wohlfahrtiimonas | MH879986 |  |  |
| ASV25 | 0.2 (0.8) | Bacteroidetes | Flavobacteriia | Flavobacteriales | Flavobacteriaceae | Apibacter | MH879894 |  |  |
| ASV61 | 0.2 (0.4) | Firmicutes | Clostridia | Clostridiales | Ruminococcaceae | Anaerotruncus | MH879928 |  |  |
| ASV117 | 0.2 (0.7) | Firmicutes | Clostridia | Clostridiales | Ruminococcaceae | Ruminiclostridium_5 | MH879983 |  |  |
| ASV99 | 0.1 (0.4) | Firmicutes | Negativicutes | Selenomonadales | Veillonellaceae |  | MH879966 |  |  |
| ASV32 | 0.1 (0.6) | Bacteroidetes | Bacteroidia | Bacteroidales | Porphyromonadaceae | Dysgonomonas | MH879901 |  |  |
| ASV125 | 0.1 (0.7) | Proteobacteria | γ-proteobacteria | Enterobacteriales | Enterobacteriaceae | Lelliottia | MH879991 |  |  |
| ASV63 | 0.1 (0.5) | Bacteroidetes | Bacteroidia | Bacteroidales | Porphyromonadaceae | Dysgonomonas | MH879930 |  |  |
| ASV76 | 0.1 (0.7) | Bacteroidetes | Bacteroidia | Bacteroidales | Porphyromonadaceae | Dysgonomonas | MH879943 |  |  |
| ASV110 | 0.1 (0.7) | Firmicutes | Bacilli | Lactobacillales | Lactobacillaceae | Lactobacillus | MH879976 |  |  |
| ASV118 | 0.1 (0.7) | Bacteroidetes | Bacteroidia | Bacteroidales | Porphyromonadaceae | Dysgonomonas | MH879984 |  |  |
| ASV43 | 0.1 (0.7) | Firmicutes | Bacilli | Lactobacillales | Streptococcaceae | Lactococcus | MH879912 |  |  |
| ASV69 | 0.1 (0.5) | Firmicutes | Clostridia | Clostridiales | Ruminococcaceae | Soleaferrea | MH879936 |  |  |
| ASV123 | 0.1 (0.4) | Firmicutes | Erysipelotrichia | Erysipelotrichales | Erysipelotrichaceae |  | MH879989 |  |  |
| ASV53 | 0.1 (0.3) | Proteobacteria | γ-proteobacteria |  |  |  | MH879920 |  |  |
| ASV57 | 0.1 (0.4) | Firmicutes | Clostridia | Clostridiales | Christensenellaceae |  | MH879924 |  |  |
| ASV90 | 0.1 (0.6) | Proteobacteria | γ-proteobacteria | Enterobacteriales | Enterobacteriaceae |  | MH879957 |  |  |
| ASV122 | 0.1 (0.4) | Firmicutes | Bacilli | Lactobacillales | Lactobacillaceae | Lactobacillus | MH879988 |  |  |
| ASV62 | 0.1 (0.2) | Bacteroidetes | Bacteroidia | Bacteroidales | Rikenellaceae |  | MH879929 |  |  |
| ASV91 | 0.1 (0.4) | Proteobacteria | γ-proteobacteria | Orbales | Orbaceae |  | MH879958 |  |  |
| ASV107 | 0.1 (0.5) | Proteobacteria | γ-proteobacteria | Xanthomonadales | Xanthomonadaceae |  | MH879973 |  |  |
| ASV84 | 0.1 (0.3) | Bacteroidetes | Bacteroidia | Bacteroidales | Porphyromonadaceae | Dysgonomonas | MH879951 |  |  |
| ASV126 | 0.1 (0.4) | Proteobacteria | γ-proteobacteria | Pseudomonadales | Pseudomonadaceae |  | MH879992 |  |  |
| ASV59 | 0.1 (0.3) | Firmicutes | Clostridia | Clostridiales | Ruminococcaceae | Ruminiclostridium_5 | MH879926 |  |  |
| ASV27 | 0.1 (0.2) | Proteobacteria | β-proteobacteria | Neisseriales | Neisseriaceae |  | MH879896 |  |  |
| ASV36 | 0.1 (0.5) | Tenericutes | Mollicutes | Entomoplasmatales | Spiroplasmataceae | Spiroplasma | MH879905 |  |  |
| ASV92 | 0.1 (0.5) | Proteobacteria | γ-proteobacteria | Enterobacteriales | Enterobacteriaceae |  | MH879959 |  |  |
| ASV108 | 0.1 (0.3) | Proteobacteria | γ-proteobacteria | Enterobacteriales | Enterobacteriaceae | Kluyvera | MH879974 |  |  |
| ASV160 | 0.1 (0.5) | Bacteroidetes | Bacteroidia | Bacteroidales | Porphyromonadaceae | Dysgonomonas | MH880023 |  |  |
| ASV139 | 0.1 (0.5) | Proteobacteria | γ-proteobacteria | Xanthomonadales | Xanthomonadaceae | Wohlfahrtiimonas | MH880004 |  |  |
| ASV135 | 0.1 (0.4) | Firmicutes | Bacilli | Lactobacillales | Carnobacteriaceae |  | MH880000 |  |  |
| ASV130 | 0.1 (0.4) | Bacteroidetes | Bacteroidia | Bacteroidales | Porphyromonadaceae | Dysgonomonas | MH879996 |  |  |
| ASV146 | 0.1 (0.4) | Proteobacteria | γ-proteobacteria | Xanthomonadales | Xanthomonadaceae |  | MH880011 |  |  |
| ASV77 | 0.1 (0.4) | Firmicutes | Bacilli | Lactobacillales | Enterococcaceae | Enterococcus | MH879944 |  |  |
| ASV148 | 0.1 (0.4) | Firmicutes | Clostridia | Clostridiales |  |  | MH880012 |  |  |
| ASV95 | 0.1 (0.3) | Bacteroidetes | Bacteroidia | Bacteroidales | Porphyromonadaceae | Dysgonomonas | MH879962 |  |  |
| ASV134 | 0.1 (0.4) | Firmicutes | Bacilli | Lactobacillales | Enterococcaceae | Enterococcus | MH879999 |  |  |
| ASV190 | 0.1 (0.3) | Proteobacteria | γ-proteobacteria | Enterobacteriales | Enterobacteriaceae | Serratia | MH880047 |  |  |
| ASV169 | 0.1 (0.3) | Firmicutes | Clostridia | Clostridiales | Ruminococcaceae | Soleaferrea | MH880032 |  |  |
| ASV144 | 0.1 (0.2) | Proteobacteria | γ-proteobacteria | Pseudomonadales | Pseudomonadaceae |  | MH880009 |  |  |
| ASV159 | 0.1 (0.3) | Firmicutes | Bacilli | Lactobacillales | Enterococcaceae | Enterococcus | MH880022 |  |  |
| ASV170 | 0.1 (0.3) | Fusobacteria | Fusobacteriia | Fusobacteriales | Leptotrichiaceae | Sebaldella | MH880033 |  |  |
| ASV138 | 0.1 (0.3) | Bacteroidetes | Bacteroidia | Bacteroidales | Porphyromonadaceae | Dysgonomonas | MH880003 |  |  |
| ASV153 | 0.1 (0.2) | Firmicutes | Bacilli | Lactobacillales | Enterococcaceae | Enterococcus | MH880016 |  |  |
| ASV155 | 0.1 (0.2) | Firmicutes | Bacilli | Lactobacillales | Enterococcaceae | Enterococcus | MH880018 |  |  |
| ASV164 | 0.1 (0.3) | Proteobacteria | γ-proteobacteria | Enterobacteriales | Enterobacteriaceae | Buttiauxella | MH880027 |  |  |
| ASV199 | 0.1 (0.3) | Proteobacteria | γ-proteobacteria | Enterobacteriales | Enterobacteriaceae | Serratia | MH880054 |  |  |
| ASV115 | 0.0 (0.2) | Bacteroidetes | Bacteroidia | Bacteroidales | Porphyromonadaceae | Dysgonomonas | MH879981 |  |  |
| ASV137 | 0.0 (0.2) | Proteobacteria | γ-proteobacteria | Orbales | Orbaceae | Schmidhempelia | MH880002 |  |  |
| ASV165 | 0.0 (0.2) | Firmicutes | Bacilli | Lactobacillales | Lactobacillaceae | Lactobacillus | MH880028 |  |  |
| ASV172 | 0.0 (0.2) | Proteobacteria | α-proteobacteria | Rhodospirillales | Acetobacteraceae |  | MH880035 |  |  |
| ASV186 | 0.0 (0.2) | Firmicutes | Bacilli | Lactobacillales | Enterococcaceae | Enterococcus | MH880044 |  |  |
| ASV150 | 0.0 (0.2) | Proteobacteria | γ-proteobacteria | Pseudomonadales | Pseudomonadaceae |  | MH880014 |  |  |
| ASV173 | 0.0 (0.2) | Actinobacteria | Actinobacteria | Micrococcales | Micrococcaceae | Pseudarthrobacter | MH880036 |  |  |
| ASV8 | 0.0 (0.1) | Proteobacteria | δ-proteobacteria | Desulfovibrionales | Desulfovibrionaceae | Desulfovibrio | MH879877 |  |  |
| ASV100 | 0.0 (0.2) | Firmicutes | Clostridia | Clostridiales | Lachnospiraceae | Tyzzerella_3 | MH879967 |  |  |
| ASV128 | 0.0 (0.1) | Firmicutes | Bacilli | Lactobacillales | Streptococcaceae | Lactococcus | MH879994 |  |  |
| ASV181 | 0.0 (0.2) | Proteobacteria | γ-proteobacteria | Enterobacteriales | Enterobacteriaceae | Enterobacter | MH880042 |  |  |
| ASV104 | 0.0 (0.1) | Proteobacteria | γ-proteobacteria | Orbales | Orbaceae |  | MH879970 |  |  |
| ASV143 | 0.0 (0.1) | Proteobacteria | γ-proteobacteria | Orbales | Orbaceae |  | MH880008 |  |  |
| ASV171 | 0.0 (0.1) | Bacteroidetes | Bacteroidia | Bacteroidales | Porphyromonadaceae | Dysgonomonas | MH880034 |  |  |
| ASV175 | 0.0 (0.1) | Proteobacteria | α-proteobacteria | Rhizobiales | Bartonellaceae | Bartonella | MH880038 |  |  |
| ASV177 | 0.0 (0.1) | Bacteroidetes | Bacteroidia | Bacteroidales | Porphyromonadaceae | Dysgonomonas | MH880039 |  |  |
| ASV121 | 0.0 (0.1) | Actinobacteria | Actinobacteria | Micrococcales | Sanguibacteraceae | Sanguibacter | MH879987 |  |  |
| ASV145 | 0.0 (0.1) | Firmicutes | Bacilli | Lactobacillales | Enterococcaceae | Enterococcus | MH880010 |  |  |
| ASV132 | 0.0 (0.1) | Proteobacteria | δ-proteobacteria | Desulfovibrionales | Desulfovibrionaceae | Desulfovibrio | MH879998 |  |  |
| ASV141 | 0.0 (0.1) | Proteobacteria | γ-proteobacteria | Orbales | Orbaceae |  | MH880006 |  |  |
| ASV179 | 0.0 (0.1) | Proteobacteria | γ-proteobacteria | Enterobacteriales | Enterobacteriaceae | Raoultella | MH880040 |  |  |
| ASV188 | 0.0 (0.1) | Proteobacteria | γ-proteobacteria | Enterobacteriales | Enterobacteriaceae |  | MH880046 |  |  |
| ASV116 | 0.0 (0.1) | Firmicutes | Clostridia | Clostridiales | Ruminococcaceae | Soleaferrea | MH879982 |  |  |
| ASV202 | 0.0 (0.1) | Proteobacteria | γ-proteobacteria | Enterobacteriales | Enterobacteriaceae | Kluyvera | MH880056 |  |  |
| ASV187 | 0.0 (0.1) | Firmicutes | Bacilli | Lactobacillales | Enterococcaceae | Enterococcus | MH880045 |  |  |
| ASV67 | 0.0 (0.1) | Firmicutes | Bacilli | Lactobacillales | Streptococcaceae | Lactococcus | MH879934 |  |  |
| ASV161 | 0.0 (0.1) | Proteobacteria | γ-proteobacteria | Enterobacteriales | Enterobacteriaceae |  | MH880024 |  |  |
| ASV197 | 0.0 (0.1) | Bacteroidetes | Bacteroidia | Bacteroidales | Rikenellaceae |  | MH880052 |  |  |
| ASV208 | 0.0 (0.1) | Proteobacteria | β-proteobacteria | Burkholderiales | Comamonadaceae | Delftia | MH880059 |  |  |
| ASV156 | 0.0 (0.1) | Proteobacteria | γ-proteobacteria | Orbales | Orbaceae |  | MH880019 |  |  |
| ASV162 | 0.0 (0.1) | Proteobacteria | γ-proteobacteria | Enterobacteriales | Enterobacteriaceae | Raoultella | MH880025 |  |  |
| ASV180 | 0.0 (0.1) | Firmicutes | Bacilli | Lactobacillales | Lactobacillaceae | Lactobacillus | MH880041 |  |  |
| ASV80 | 0.0 (0.1) | Bacteroidetes | Bacteroidia | Bacteroidales | Porphyromonadaceae | Dysgonomonas | MH879947 |  |  |
| ASV113 | 0.0 (0.1) | Firmicutes | Erysipelotrichia | Erysipelotrichales | Erysipelotrichaceae | Breznakia | MH879979 |  |  |
| ASV184 | 0.0 (0.1) | Bacteroidetes | Bacteroidia | Bacteroidales | Porphyromonadaceae | Dysgonomonas | MH880043 |  |  |
| ASV214 | 0.0 (0.1) | Firmicutes | Bacilli | Bacillales | Bacillaceae | Bacillus | MH880061 |  |  |
| ASV232 | 0.0 (0.1) | Firmicutes | Clostridia | Clostridiales | Family_XI | Gallicola | MH880070 |  |  |
| ASV191 | 0.0 (0.1) | Proteobacteria | γ-proteobacteria | Pseudomonadales | Pseudomonadaceae | Pseudomonas | MH880048 |  |  |
| ASV217 | 0.0 (0.1) | Bacteroidetes | Bacteroidia | Bacteroidales | Porphyromonadaceae | Dysgonomonas | MH880063 |  |  |
| ASV34 | 0.0 (0.1) | Bacteroidetes | Bacteroidia | Bacteroidales | Porphyromonadaceae | Dysgonomonas | MH879903 |  |  |
| ASV127 | 0.0 (0.1) | Proteobacteria | γ-proteobacteria | Orbales | Orbaceae | Schmidhempelia | MH879993 |  |  |
| ASV166 | 0.0 (0.1) | Proteobacteria | γ-proteobacteria | Orbales | Orbaceae | Gilliamella | MH880029 |  |  |
| ASV198 | 0.0 (0.1) | Actinobacteria | Actinobacteria | Micrococcales | Incertae_Sedis | Luteimicrobium | MH880053 |  |  |
| ASV239 | 0.0 (0.0) | Proteobacteria | α-proteobacteria | Caulobacterales | Caulobacteraceae | Brevundimonas | MH880072 |  |  |
| ASV97 | 0.0 (0.0) | Bacteroidetes | Bacteroidia | Bacteroidales | Porphyromonadaceae | Dysgonomonas | MH879964 |  |  |
| ASV213 | 0.0 (0.0) | Firmicutes | Bacilli | Lactobacillales | Lactobacillaceae | Lactobacillus | MH880060 |  |  |
| ASV248 | 0.0 (0.0) | Proteobacteria | γ-proteobacteria |  |  |  | MH880076 |  |  |
| ASV54 | 0.0 (0.0) | Firmicutes | Bacilli | Lactobacillales | Lactobacillaceae | Lactobacillus | MH879921 |  |  |
| ASV93 | 0.0 (0.0) | Bacteroidetes | Flavobacteriia | Flavobacteriales | Flavobacteriaceae | Apibacter | MH879960 |  |  |
| ASV216 | 0.0 (0.0) | Bacteroidetes | Bacteroidia | Bacteroidales | Porphyromonadaceae | Dysgonomonas | MH880062 |  |  |
| ASV192 | 0.0 (0.0) | Firmicutes | Bacilli | Lactobacillales | Lactobacillaceae | Lactobacillus | MH880049 |  |  |
| ASV206 | 0.0 (0.0) | Proteobacteria | γ-proteobacteria | Orbales | Orbaceae | Gilliamella | MH880058 |  |  |
| ASV218 | 0.0 (0.0) | Actinobacteria | Actinobacteria | Micrococcales | Micrococcaceae | Pseudarthrobacter | MH880064 |  |  |
| ASV236 | 0.0 (0.0) | Bacteroidetes | Bacteroidia | Bacteroidales | Porphyromonadaceae | Dysgonomonas | MH880071 |  |  |
| ASV251 | 0.0 (0.0) | Actinobacteria | Actinobacteria | Corynebacteriales | Nocardiaceae | Rhodococcus | MH880078 |  |  |
