# Supplementary material for: Bacterial Associates of a Gregarious Riparian Beetle With Explosive Defensive Chemistry
Source: Front Microbiol. 2018 Oct 5;9:2361. doi: 10.3389/fmicb.2018.02361 (PMC6182187; doi:10.3389/fmicb.2018.02361)
Supplement: Supplementary file 12 [file Table_4.docx]

**Table S4**: **Taxonomic identities of the top 10 amplicon sequence variants present in MGMT samples from Site 1 (Madera Canyon, AZ).** “Avg. (STD)” is the average relative abundance per individual with the standard deviation in parentheses. Taxonomy was assigned using RDP classifier against the Silva taxonomic training set.

| **ASVid** | **Avg. (STD)** | **Phylum** | **Class** | **Order** | **Family** | **Genus** | **Accession #** |  |
| --- | --- | --- | --- | --- | --- | --- | --- | --- |
| ASV3 | 12.8 (12.7) | Firmicutes | Bacilli | Lactobacillales | Enterococcaceae | Enterococcus | MH879872 |  |
| ASV1 | 12.1 (29.7) | Tenericutes | Mollicutes | Entomoplasmatales | Spiroplasmataceae | Spiroplasma | MH879870 |  |
| ASV2 | 10.2 (16.0) | Tenericutes | Mollicutes | Entomoplasmatales | Spiroplasmataceae | Spiroplasma | MH879877 |  |
| ASV8 | 7.8 (19.1) | Proteobacteria | Deltaproteobacteria | Desulfovibrionales | Desulfovibrionaceae | Desulfovibrio | MH879912 |  |
| ASV43 | 5.2 (12.6) | Firmicutes | Bacilli | Lactobacillales | Streptococcaceae | Lactococcus | MH879912 |  |
| ASV13 | 5.1 (9.9) | Firmicutes | Bacilli | Lactobacillales | Lactobacillaceae | Lactobacillus | MH879882 |  |
| ASV12 | 3.7 (8.7) | Proteobacteria | γ-proteobacteria | Pseudomonadales | Pseudomonadaceae |  | MH879881 |  |
| ASV5 | 3.7 (6.2) | Tenericutes | Mollicutes | Entomoplasmatales | Spiroplasmataceae | Spiroplasma | MH879874 |  |
| ASV16 | 3.5 (5.0) | Fusobacteria | Fusobacteriia | Fusobacteriales | Leptotrichiaceae | Sebaldella | MH879885 |  |
| ASV54 | 3.4 (8.4) | Firmicutes | Bacilli | Lactobacillales | Lactobacillaceae | Lactobacillus | MH879921 |  |
