# Supplementary material for: Bacterial Associates of a Gregarious Riparian Beetle With Explosive Defensive Chemistry
Source: Front Microbiol. 2018 Oct 5;9:2361. doi: 10.3389/fmicb.2018.02361 (PMC6182187; doi:10.3389/fmicb.2018.02361)
Supplement: Supplementary file 13 [file Table_5.docx]

**Table S5**: **Taxonomic identities of the top 10 amplicon sequence variants present in ileum samples from Site 1 (Madera Canyon, AZ).** “Avg. (STD)” is the average relative abundance per individual with the standard deviation in parentheses. Taxonomy was assigned using RDP classifier against the Silva taxonomic training set.

| **ASVid** | **Avg. (STD)** | **Phylum** | **Class** | **Order** | **Family** | **Genus** | **Accession #** |  |
| --- | --- | --- | --- | --- | --- | --- | --- | --- |
| ASV6 | 6.0 (6.1) | Firmicutes | Bacilli | Lactobacillales |  |  | MH879875 |  |
| ASV3 | 5.8 (2.5) | Firmicutes | Bacilli | Lactobacillales | Enterococcaceae | Enterococcus | MH879872 |  |
| ASV19 | 5.5 (4.6) | Proteobacteria | γ-proteobacteria | Enterobacteriales | Enterobacteriaceae |  | MH879888 |  |
| ASV9 | 5.3 (7.0) | Bacteroidetes | Bacteroidia | Bacteroidales | Porphyromonadaceae | Dysgonomonas | MH879878 |  |
| ASV27 | 4.5 (3.6) | Proteobacteria | Betaproteobacteria | Neisseriales | Neisseriaceae |  | MH879896 |  |
| ASV23 | 3.9 (3.8) | Bacteroidetes | Bacteroidia | Bacteroidales | Porphyromonadaceae | Dysgonomonas | MH879892 |  |
| ASV22 | 3.5 (3.4) | Proteobacteria | γ-proteobacteria | Enterobacteriales | Enterobacteriaceae |  | MH879891 |  |
| ASV14 | 3.0 (3.2) | Firmicutes | Clostridia | Clostridiales |  |  | MH879883 |  |
| ASV25 | 3.0 (3.2) | Bacteroidetes | Flavobacteriia | Flavobacteriales | Flavobacteriaceae | Apibacter | MH879894 |  |
| ASV20 | 3.0 (2.2) | Firmicutes | Bacilli | Lactobacillales |  |  | MH879889 |  |
