# Supplementary material for: Bacterial Associates of a Gregarious Riparian Beetle With Explosive Defensive Chemistry
Source: Front Microbiol. 2018 Oct 5;9:2361. doi: 10.3389/fmicb.2018.02361 (PMC6182187; doi:10.3389/fmicb.2018.02361)
Supplement: Supplementary file 14 [file Table_6.docx]

**Table S6**: **Taxonomic identities of the top 10 amplicon sequence variants present in the secretory cell samples from Site 1 (Madera Canyon, AZ)**. (STD)” is the average relative abundance per individual with the standard deviation in parentheses. Taxonomy was assigned using RDP classifier against the Silva taxonomic training set.

| **ASVid** | **Avg. (STD)** | **Phylum** | **Class** | **Order** | **Family** | **Genus** | **Accession #** |  |
| --- | --- | --- | --- | --- | --- | --- | --- | --- |
| ASV18 | 11.6 (28.5) | Proteobacteria | γ-proteobacteria | Enterobacteriales | Enterobacteriaceae | Pragia | MH879887 |  |
| ASV6 | 8.8 (12.9) | Firmicutes | Bacilli | Lactobacillales |  |  | MH879875 |  |
| ASV9 | 7.3 (13.0) | Bacteroidetes | Bacteroidia | Bacteroidales | Porphyromonadaceae | Dysgonomonas | MH879878 |  |
| ASV3 | 6.2 (8.7) | Firmicutes | Bacilli | Lactobacillales | Enterococcaceae | Enterococcus | MH879872 |  |
| ASV25 | 4.3 (8.8) | Bacteroidetes | Flavobacteriia | Flavobacteriales | Flavobacteriaceae | Apibacter | MH879894 |  |
| ASV32 | 3.7 (6.4) | Bacteroidetes | Bacteroidia | Bacteroidales | Porphyromonadaceae | Dysgonomonas | MH879901 |  |
| ASV4 | 3.6 (8.5) | Proteobacteria | γ-proteobacteria | Pseudomonadales | Pseudomonadaceae |  | MH879873 |  |
| ASV2 | 3.5 (5.9) | Tenericutes | Mollicutes | Entomoplasmatales | Spiroplasmataceae | Spiroplasma | MH879871 |  |
| ASV5 | 3.1 (6.3) | Tenericutes | Mollicutes | Entomoplasmatales | Spiroplasmataceae | Spiroplasma | MH879874 |  |
| ASV15 | 3.0 (6.0) | Proteobacteria | γ-proteobacteria | Pseudomonadales | Pseudomonadaceae |  | MH879884 |  |
