# Supplementary material for: Bacterial Associates of a Gregarious Riparian Beetle With Explosive Defensive Chemistry
Source: Front Microbiol. 2018 Oct 5;9:2361. doi: 10.3389/fmicb.2018.02361 (PMC6182187; doi:10.3389/fmicb.2018.02361)
Supplement: Supplementary file 15 [file Table_7.docx]

**Table S7**: **Taxonomic identities of the top 10 amplicon sequence variants from MGMT samples from Site 2 (Happy Valley, AZ).** “Avg. (STD)” is the average relative abundance per individual with the standard deviation in parentheses. Taxonomy was assigned using RDP classifier against the Silva taxonomic training set.

| **ASVid** | **Avg. (STD)** | **Phylum** | **Class** | **Order** | **Family** | **Genus** | **Accession #** |
| --- | --- | --- | --- | --- | --- | --- | --- |
| ASV2 | 41.7 (41.8) | Tenericutes | Mollicutes | Entomoplasmatales | Spiroplasmataceae | Spiroplasma | MH879871 |
| ASV1 | 20.0 (44.7) | Tenericutes | Mollicutes | Entomoplasmatales | Spiroplasmataceae | Spiroplasma | MH879870 |
| ASV5 | 9.2 (20.6) | Tenericutes | Mollicutes | Entomoplasmatales | Spiroplasmataceae | Spiroplasma | MH879874 |
| ASV12 | 8.7 (19.5) | Proteobacteria | γ-proteobacteria | Pseudomonadales | Pseudomonadaceae |  | MH879881 |
| ASV34 | 3.1 (7.0) | Bacteroidetes | Bacteroidia | Bacteroidales | Porphyromonadaceae | Dysgonomonas | MH879903 |
| ASV22 | 3.0 (6.7) | Proteobacteria | γ-proteobacteria | Enterobacteriales | Enterobacteriaceae |  | MH879891 |
| ASV29 | 2.3 (5.2) | Proteobacteria | γ-proteobacteria | Pseudomonadales | Pseudomonadaceae | Pseudomonas | MH879898 |
| ASV11 | 2.2 (4.5) | Proteobacteria | γ-proteobacteria | Orbales | Orbaceae |  | MH879880 |
| ASV7 | 1.8 (3.8) | Proteobacteria | γ-proteobacteria | Orbales | Orbaceae |  | MH879876 |
| ASV73 | 0.9 (2.0) | Proteobacteria | γ-proteobacteria | Pseudomonadales | Pseudomonadaceae |  | MH879940 |
