# Supplementary material for: Bacterial Associates of a Gregarious Riparian Beetle With Explosive Defensive Chemistry
Source: Front Microbiol. 2018 Oct 5;9:2361. doi: 10.3389/fmicb.2018.02361 (PMC6182187; doi:10.3389/fmicb.2018.02361)
Supplement: Supplementary file 16 [file Table_8.docx]

**Table S8**: **Taxonomic identities of the top 10 amplicon sequence variants from ileum samples from Site 2 (Happy Valley, AZ).** “Avg. (STD)” is the average relative abundance per individual with the standard deviation in parentheses. Taxonomy was assigned using RDP classifier against the Silva taxonomic training set.

| **ASVid** | **Avg. (STD)** | **Phylum** | **Class** | **Order** | **Family** | **Genus** | **Accession #** |  |
| --- | --- | --- | --- | --- | --- | --- | --- | --- |
| ASV7 | 14.5 (17.7) | Proteobacteria | γ-proteobacteria | Orbales | Orbaceae |  | MH879876 |  |
| ASV4 | 9.6 (17.7) | Proteobacteria | γ-proteobacteria | Pseudomonadales | Pseudomonadaceae |  | MH879873 |  |
| ASV39 | 6.0 (13.4) | Bacteroidetes | Flavobacteriia | Flavobacteriales | Flavobacteriaceae | Apibacter | MH879908 |  |
| ASV38 | 5.8 (13.0) | Proteobacteria | Betaproteobacteria | Neisseriales | Neisseriaceae |  | MH879907 |  |
| ASV14 | 5.2 (6.4) | Firmicutes | Clostridia | Clostridiales |  |  | MH879883 |  |
| ASV20 | 4.3 (8.3) | Firmicutes | Bacilli | Lactobacillales |  |  | MH879889 |  |
| ASV12 | 3.5 (6.4) | Proteobacteria | γ-proteobacteria | Pseudomonadales | Pseudomonadaceae |  | MH879881 |  |
| ASV34 | 3.0 (6.6) | Bacteroidetes | Bacteroidia | Bacteroidales | Porphyromonadaceae | Dysgonomonas | MH879903 |  |
| ASV11 | 2.8 (4.9) | Proteobacteria | γ-proteobacteria | Orbales | Orbaceae |  | MH879880 |  |
| ASV22 | 2.8 (4.6) | Proteobacteria | γ-proteobacteria | Enterobacteriales | Enterobacteriaceae |  | MH879891 |  |
